# Limited transmission of avian influenza viruses, avulaviruses, coronaviruses and *Chlamydia* sp. at the interface between wild birds and a free-range duck farm

**DOI:** 10.1186/s13567-025-01466-3

**Published:** 2025-02-08

**Authors:** Chloé Le Gall-Ladevèze, Benjamin Vollot, Julien Hirschinger, Laëtitia Lèbre, Rachid Aaziz, Karine Laroucau, Jean-Luc Guérin, Mathilde Paul, Julien Cappelle, Guillaume Le Loc’h

**Affiliations:** 1 IHAP, ENVT, INRAE, Université de Toulouse, Toulouse, France; 2 BV Nat, Aigues-Vives, France; 3 Bacterial Zoonoses Unit, Animal Health Laboratory, University Paris-Est, Anses, Maisons-Alfort, France; 4 ASTRE, CIRAD, INRAE, Université de Montpellier, Montpellier, France; 5 CIRAD, UMR ASTRE, 34398 Montpellier, France

**Keywords:** Commensal birds, free-range poultry, environment, AIV, avulavirus, coronavirus, *Chlamydia* sp., wild-domestic interface

## Abstract

Recent outbreaks of highly pathogenic avian influenza in Europe have raised questions regarding the epidemiological role of commensal wild birds on free-range poultry farms. This study aimed to assess the prevalence of avian influenza viruses (AIV), avulaviruses, coronaviruses and *Chlamydia* sp. in commensal wild birds on a free-range duck farm in southwestern France and to evaluate possible transmission events at the wild‒domestic interface. From 2019 through 2021, a longitudinal study was conducted on wild birds, domestic ducks and their shared environment on farms. Commensal wild birds were captured and sampled for blood and swabs, and fresh feces from cattle egrets visiting the farm were collected. In parallel, domestic ducks were sampled, and environmental samples were collected. The presence of the four pathogens was tested by q(RT-)PCR, and the immunity of wild birds to AIV and Newcastle disease virus (NDV) was tested by ELISA. Wild birds were found to shed AIV and *Chlamydia* only, with a low prevalence (< 3%). The seroprevalence rates were less than 10% for AIV and less than 4.5% for NDV. No significant temporal trend was identified. Ducks and their environment frequently test simultaneously positive for the same pathogens (19 to 44% of flocks), mostly during fall‒winter. In addition to unrelated temporal patterns, the identification of pathogens in wild birds seemed unrelated to that in domestic ducks. These results suggest a low transmissibility of the avian pathogens tested in our study at the wild‒domestic interface and highlight the limited contribution of commensal wild birds in comparison with free-range poultry to the global microbiological pressure on the environment.

**Supplementary Information:**

The online version contains supplementary material available at 10.1186/s13567-025-01466-3.

## Introduction

The emergence, re-emergence and spread of animal pathogenic infectious agents have increased over recent decades, which may be related mostly to human activities, particularly agricultural practices and environmental modifications that favour contact between wildlife and livestock [1]. Among emerging agents, avian influenza viruses (AIV) present zoonotic and highly pathogenic strains (HPAIV) with broad mutation and reassortment abilities, enabling shifts in host range and pathogenicity [2]. The natural reservoirs of AIV are water birds (Anseriformes, Charadriiformes) [3], including numerous long-distance migratory species that can host and spread viruses between continents [4, 5].

Southwest France, particularly the region’s duck production sector, has been deeply affected by three highly pathogenic avian influenza (HPAI) epizootics in recent years [6–8]. The region is rich in free-range farms that breed Anseriformes (ducks and geese) and Galliformes (chicken, guinea fowl, quail). Free-range duck farms in particular have been shown to increase the risk of HPAIV introduction at a regional scale [9]. As few contacts between free-ranging ducks and wild waterfowl are observed in the region but intense contacts are observed with some species of terrestrial commensal birds [10], the role of the latter in HPAIV transmission has come under scrutiny [11], especially since the susceptibility of some terrestrial commensal species to AIV has been demonstrated [12, 13]. Thus, such species could play a role in the epidemiology of AIV, if not as maintenance hosts and then as bridge hosts [14, 15], contributing to viral spread between farms or to spillover and spillback events with wetlands and their water bird populations.

In addition to AIV, other avian pathogens pose health risks to free-range duck farms and can have wide host ranges, allowing them to potentially circulate at the wild‒domestic bird interface. Among pathogens infecting domestic ducks, viruses of the *Avulavirinae* subfamily (including Newcastle disease virus) present various genotypes that are detected in a very broad range of bird species and sometimes at the wild-domestic interface [16–18]. Similarly, viruses of the *Orthocoronavirinae*, a subfamily belonging to the *Gammacoronavirus* (including Infectious bronchitis virus) and *Deltacoronavirus* genera, can also infect a wide variety of birds and can be found at the wild-domestic interface [19, 20]. In addition to these two viral taxa, bacteria of the genus *Chlamydia* are similarly broadly distributed in species and geography, with high asymptomatic prevalence values of *Chlamydia psittaci* observed in wild and domestic ducks [21–23]. In addition to *C. psittaci*, other *Chlamydia* species are harbored by birds. Of these, *C. abortus*, a species that also includes avian strains alongside well-known ruminant strains [24], is a recently described species in wild avifauna [25–27].

*Avulavirinae* (avulaviruses), *Orthocoronavirinae* (coronaviruses) and *Chlamydia* sp. follow transmission routes that are similar to those of AIV. All four pathogens may thus follow similar epidemiological patterns [28] and be shared through the same interactions at the wild-domestic interface, thus being potential transmission markers of each other.

The aim of this study was to evaluate the possibility of pathogen spread in biotic (wild and domestic birds) and abiotic (environmental) compartments at the wild-domestic interface on a typical free-range duck farm in southwestern France. For this purpose, a longitudinal monitoring and characterization study of AIV and three groups of avian pathogens was implemented, focusing in parallel on commensal wild birds, domestic duck flocks, and the environments that they share (the ducks’ outdoor foraging areas). The findings of the present study should improve the understanding of the epidemiological mechanisms of these avian pathogens in local host communities found on duck farms, helping to better understand how to manage the risks of spillover and spillback between wild birds and poultry.

## Materials and methods

### Study site

The study was implemented on a typical duck farm in the department of Gers, Southwest France. The farm environment and bird community were described in a previous study [10]. The farm breeds mule ducks under the “Canard à Foie Gras du Sud-Ouest” label, which requires at least 14 weeks of unlimited outdoor access. It is composed of two small 0.5 ha outdoor foraging areas for ducklings (one day to one month of age) and eight large 1.5 ha foraging areas for growing ducks (one month to 14–16 weeks of age). Like an increasing number of poultry farms in the region, an agroforestry program is implemented on the farm, so trees for wood are planted on all outdoor foraging areas, and hedges of fruit trees are planted around some of the areas. These facilities can attract wild birds that potentially contact farmed animals.

### Sample collection

#### Sampling of wild birds

From July 2019 to March 2021, 11 mist-net capture sessions were performed around outdoor areas of the farm (July, August, October and November 2019; January, February, May, November and December 2020; February and March 2021). Each session lasted three days and targeted small- to medium-sized wild bird species. Each captured bird was banded with a coded metal ring from the Muséum National d’Histoire Naturelle (MNHN), Paris, France, for individual identification. Captures and manipulations were performed by a professional bird-bander and a veterinarian following the veterinary practices set down in EU Directive 2010/63/EU for animal experiments, and the protocol was subjected to mandatory ethical approval and legal authorization by the MNH (program number 1035). A visual clinical inspection was performed, and swabs (oropharyngeal and cloacal) were collected, as were 100 µL of blood from healthy birds above 11 g (i.e., less than 10% of their blood volume) (Additional file 1 and Table 1). The swabs were stored in microtubes with 300 µL of sterile 1% phosphate-buffered saline (PBS) and kept at + 4 °C before being frozen at -80 °C when they were in the laboratory (at most 3 days after sampling). The blood was stored in sterile microtubes for 1–3 h at + 20 °C in an isolated box and then kept at + 4 °C before the serum was extracted when it was stored in the laboratory (at most 3 days later).

**Table 1:** **Distribution of samples and positive type of sample**

Year	Month	Week	Swabs from wild birds	Feces from cattle egrets	Positive ones	Blood from wild birds	Positive ones	Duck flocks	Identification of flocks sampled	Positive ones	Environment of duck areas	Identification of corresponding flocks	Positive results
2019	April	**17**						1	A				
	June	**23**						1 *	B				
		**24**						2 *	C; D	2 AIV			
	July	**30**	184		1 AIV (SYLATR) 1 Chlam (PASM)								
	August	**35**	256		2 AIV (PASD, MUS) 2 Chlam(AEG, SYL)	121	3 AIV (LUSMEG, FICUCA, SYLATR)						
	September	**37**						1 *	E				
		**39**						1 *	F				
	October	**43**	240	30	1 AIV (MOTALB) 3 Chlam(2 MOTALB, SYLATR)	142	4 AIV (2 SYLATR, TURPHI, MOTALB)				4	**G**; H; I; J	1 coronavirus (H)
	November	**45**						1	**G**	1 coronavirus			
		**48**	184	30	2 AIV (PASD, ANT)	75					2	**K**; L	
	December	**52**						1	**K**	1 AIV 1 Chlam 1 coronavirus			
2020	January	**5**	225		2 AIV (SYLATR, FRI)	112	1 AIV (PASM) 1 NDV (TURMER)				2	**M**; **N**	2 AIV 2 Chlam
	February	**8**						1 + 1*	**M**; **N**	2 AIV 1 Chlam(**M**)			
		**9**	141		2 Chlam(AEG, FRI)	88	1 AIV (PASM) 1 NDV (TURMER)				4	**M**; **N**; O; P	1 AIV (**M**) 1 Chlam(O)
	May	**19**						1 *	**Q**				
		**22**	168			140	4 AIV (LUSMEG, 2 SYLATR, TURPHI)				2	**Q**; R	
	July	**28**						1	S				
	November	**46**	225		1 Chlam(AEG PHYCOL)	147	6 AIV (2 ERIRUB, 2 PRU, PASD, TURPHI)				4	**T**; **U**; **V**; **W**	2 AIV (**T**; **U**) 2 Chlam(**T**; **U**) 2 coronavirus (**T**; **V**)
	December	**51**	47	70		40	3 AIV (3 MOTALB) 2 NDV (ANTPRA, MOTALB)				4	**T**; **U**; **V**; **W**	3 AIV (**T**; **V**; **W**) 4 Chlam
2021	February	**6**		25									
		**9**	51	52	1 AIV (BUB)	40	3 AIV (PRU, ACC, MOTALB)						
	March	**12**	10			5							

* next to some duck flocks indicate that only the results of regulatory AIV surveillance were available. The flocks are identified by letters and are shown in bold when they are sampled several times, either by swabs or environmental samples of their outdoor foraging area. For each pathogen detected, positive flocks or wild bird groups (or species if known) are indicated between brackets when necessary. Species and groups of wild birds are coded as detailed in Additional file 1. AIV stands for avian influenza virus, Avu for avulaviruses, CoV for coronaviruses and Chlam for *Chlamydia* sp.

Cattle egrets (*Bubulcus ibis*), which were observed on the farm (up to 200 birds) in winter, were sampled on five occasions during the winters of 2019–2020 and 2020–2021 at their resting site, which was located 2 km away from a small pond (October and November 2019, December 2020, twice in February 2021). During the day, when they were away foraging, a large disinfected plastic tarp was installed under their roosts. The following morning, individual fresh feces produced during the night were collected on the tarp using sterile swabs and then stored as other swabs.

#### Sampling of duck flocks

For French regulatory AIV surveillance of duck farms, 20 ducks from each flock of 10 weeks of age were randomly selected for swab sampling (tracheal and cloacal) by the veterinarian of the farm, according to the Decree of February 8th 2016, regulating biosecurity and surveillance measures against avian influenza in poultry [29]. This regulatory surveillance sampling occurred on 10 occasions (April 2019, twice in June 2019, twice in September 2019, November and December 2019, February, May and July 2020), representing 12 flocks of ducks. On five occasions (in April, November and December 2019, February and July 2020), duplicate swabs were collected by the veterinarian. Dry swabs were stored in microtubes with 300 µL of sterile 1X PBS at −80 °C when they were collected in the laboratory.

#### Sampling of environment

On the occasion of wild bird capture (during the same days), from October 2019 to December 2020, environmental samples were collected from outdoor duck foraging areas where ducks were present. For each area, a total of 50 mL of surface water was collected in a sterile tube; the samples were taken from several water puddles scattered across the area (40 mL) and 10 mL from 2 to 3 open-air drinkers. In addition, dry horizontal surfaces apparently free from duck feces but accessible to wild birds were sampled using sterile gloves and sterile wipes of gauze moistened with sterile 1X PBS. After sampling, the wipes were rolled into sterile plastic tubes with 20 mL of sterile 1X PBS. The environmental samples were systematically associated with negative controls containing only the sampling material and PBS. The environmental samples were stored at + 4 °C in the field and then frozen at −80 °C in the laboratory.

### Detection and identification of avian pathogens

#### Preparation of environmental samples

To separate extracellular nucleic acids from particles of soil and organic matter that may inhibit PCR reactions, and to concentrate the expected genetic material from large volume samples, environmental samples were prepared following a specific protocol (Additional file 2). The resulting supernatants of such cleared and concentrated samples were subsequently processed for nucleic acid extraction.

#### Nucleic acid extraction

Both RNA and DNA extractions from swabs and environmental samples were performed from their supernatants following the protocol described in Additional file 2. Swabs were pooled for screening by similar host (species or family), sample type (oral, tracheal or cloacal), and period of sampling (5 samples in each pool whenever possible). The environmental samples were processed by pools of samples from the same period and location (water and wipes from the same foraging area).

#### Optimization of broad range real-time PCR protocols

For the detection of avulaviruses, coronaviruses and *Chlamydia* sp., broad-range one-step real-time RT-PCR and real-time PCR were adapted and optimized from protocols previously described in the literature (Additional file 3).

#### Molecular detection and identification of avian pathogens

All reactions were processed on duplicate samples as detailed in Additional file 3. For each run of real-time PCR or RT-PCR, quantification and sensitivity were assessed by ten-fold dilutions of a plasmid construct including the target sequence, from 10 to 10^3^ sequence copies/µL. When a pool of swabs was positive, the corresponding individual samples were analysed following the same protocol. For all PCR, positive, negative, endogenous and exogeneous controls were used.

### AIV

One-step real-time RT-PCR targeting the M gene of all AIV was performed following a SYBR Green protocol with M52C/M253R primers was applied [30]. Positive and suspect samples for M gene real-time RT-PCR were subjected to conventional one-step RT-PCR using Bm-HA-1/Bm-NS-890R primers targeting the whole hemagglutinin (HA) sequence of AIV [31], as detailed in Additional file 3 (Table C, Identification run 1). The resulting amplified products of the expected size (1800 bp) were sequenced by Sanger technology, visualized using BioEdit version 7.0.5.3 [32] and compared with all AIV sequences available in the GenBank database using the BLAST online tool [33].

H6 specific one-step real-time RT-PCR also was performed as this subtype previously identified in duck samples from farms. The primer pair designed and used was H6-928/H6-1251, and the protocol followed is detailed in Additional file 3 (Table C, Identification run 3).

In duck flocks sampled for regulatory AIV surveillance, the standard process of identification was followed by the departmental laboratory and National Reference Laboratory (NRL) for AIV at Anses, Ploufragan, France, according to the Decree of February 8th, 2016, regulating biosecurity and surveillance measures against avian influenza in poultry [29]. M gene-positive flocks were subjected to specific real-time RT-PCR for both the H5 and H7 subtypes. Positive samples for this second test were then sequenced to obtain precise HA and NA subtype identification.

### Avulaviruses

The adapted one-step real-time RT-PCR protocol targeting the L gene of all avulaviruses with the AVU-RUB-F1/AVU-RUB-R primers [34] was applied to all samples in duplicate, as detailed in Additional file 3. Positive and suspect samples for the L gene were confirmed by a second run of semi-nested conventional PCR with the primers AVU-RUB-F2/AVU-RUB-R [34], as detailed in Additional file 3. The resulting amplified products of the expected size (200 bp) were sequenced by Sanger technology, visualized using BioEdit version 7.0.5.3 [32] and compared with all avulaviruses reference sequences using the BLAST online tool [33].

### Coronaviruses

The adapted one-step real-time RT-PCR protocol targeting the polymerase gene of all coronaviruses with AC-CoV-F/AC-CoV-R primers [35] was applied to all samples in duplicate, as detailed in Additional file 3. Positive and suspect samples for the polymerase gene were confirmed by a second run of semi-nested conventional PCR with the same primers, as detailed in Additional file 3. The resulting amplified products of the expected size (600 bp) were sequenced by Sanger technology, visualized using BioEdit version 7.0.5.3 [32] and compared with all coronaviruses sequences available in the GenBank database using the BLAST online tool [33].

#### *Chlamydia* sp.

The adapted real-time PCR protocol targeting the 23S ribosomal gene of all *Chlamydia* sp. with the Ch23S-F/Ch23S-R primers [36] was applied to all samples in duplicate, as detailed in Additional file 3. Positive and suspect PCR products were confirmed on an agarose gel electrophoresis. If bands of the expected size (180 bp) appeared, specific real-time PCR methods for *C. psittaci* and *C. abortus* were conducted on positive samples [25]. The samples identified as *C. psittaci* were then subjected to high-resolution melting (HRM)-PCR analysis for broad genotyping as previously described [37], which was doubled with Sanger sequencing of the *ompA* gene. The samples identified as *C. abortus* were genotyped on the basis of their plasmid sequence and by multilocus sequence typing (MLST) on seven targets, as described in previous studies [25].

#### Serologies for AIV and NDV

The blood samples were subsequently centrifuged at 2500 RCF for 5 min at + 4 °C to separate the serum from the cell mixture. ELISA serologies for AIV and NDV were performed on 10 µL of sera using ID Screen^®^ Influenza A Antibody Competition Multispecies (FLUACA) kit and an ID Screen^®^ Newcastle Disease Competition (NDVC) kit (Innovative Diagnostics, Grabels, France), respectively, following the manufacturers’ instructions. Samples were considered positive when the competition percentage was less than 50% for FLUACA or 70% for NDVC, that is, when they were suspected or positive according to the manufacturer, because these kits were not initially optimized for wild birds [38]. If enough serum remained from the AIV seropositive samples, the H5 subtype was tested with the ID Screen^®^ Influenza H5 Antibody Competition (FLUACH5) kit (Innovative Diagnostics). The samples were considered positive when the percentage of competition was less than 60% (positive or suspect according to the manufacturer’s instructions).

### Epidemiological and statistical analyses

In wild birds, individual molecular analysis of positive pools in some cases does not allow the retrieval of positive individual(s). However, as successful individual analyses lead to one positive individual per positive pool, each positive pool of wild birds was considered to come from only one individual. A pool of individuals was considered positive when the pool of cloacal or oropharyngeal swabs was positive. This approximation of individual to pooled prevalence was considered realistic because of the low number of positives among the large number of samples [39]. For duck flocks and environmental samples, as the number of samples was irrespective of the size of the flock, the results were analysed considering flocks or the environment as a single epidemiological unit. A duck flock was then considered positive when either one cloacal or oropharyngeal swab was positive. Similarly, the environment of a flock was considered positive when its corresponding pooled environmental sample was positive.

The observed prevalence values were corrected for the estimated sensitivity (Se) and specificity (Sp) of the diagnostic tests used. The Se of the influenza A ELISA kit was estimated to be 0.9, and its Sp was 0.9 in the wild birds sampled for the present study [38]. The Se of the NDV ELISA kit was estimated to be 1, and its Sp was 0.97 [40]. As real-time PCR and RT-PCR tests are typically performed on both oropharyngeal and cloacal swabs from the same individual bird (increasing Se), with a strict confirmation of the results and a low detection threshold in duplicate, Se was considered to be 0.99 and Sp 1. Estimations of true PCR prevalence and confidence intervals were calculated for each sample via the Epitools “Estimated true prevalence” online tool [41], following methods from Rogan & Gladen [42] and Reiczigel et al. [43], respectively. For serological prevalence, only a Wilson confidence interval on apparent prevalence was calculated following methods from Brown et al. [44], as the estimation of true prevalence was not adapted to low numbers. The resulting prevalence values were numerically compared using R software version 4.0.5 [45], and differences between groups of wild birds and between sampling times were statistically evaluated using chi-square tests.

## Results

### Sample collection

#### Sampling of wild birds

Between July 2019 and March 2021, the 11 capture sessions resulted in the collection of an average of 157 birds per session, with a median of 184, a minimum of 10 (due to one shorter session lasting 24 h) and a maximum of 256 (Table 1). In total, 1731 wild birds were captured and swabbed by oropharyngeal and cloacal routes. Blood was collected from 910. The captured birds represented 62 species representing 30 families and 10 orders and were divided for the analyses into 26 groups of similar life traits and related taxa (Additional file 1).

In addition to live bird swabs, a total of 207 cattle egret fecal samples were collected at night. These samples were distributed on five occasions: in October and November 2019, in December 2020 and February 2021, simultaneously with wild bird captures, and on a separate additional occasion in February 2021 (Table 1). An average of 41 feces were collected at each time point, with a median of 30, a minimum of 25 and a maximum of 70.

#### Sampling of duck flocks

On 10 occasions between April 2019 and July 2020, a total of 240 ducks from 12 flocks (20 ducks per flock) were swabbed by tracheal and cloacal routes, 7 of which were only subjected to official AIV surveillance (Table 1).

#### Sampling of environment

Environmental samples from outdoor duck areas were collected on seven occasions simultaneously with wild bird captures between October 2019 and December 2020 (Table 1). A total of 22 areas were sampled, corresponding to 16 different flocks, of which 5 flocks were also sampled by swabs.

### Detection and identification of pathogens in wild birds

#### AIV in wild birds

Overall, the M gene was detected in 9 individual (or pooled) wild bird samples out of 1938 individuals (0.46% corrected, 95% CI: 0.2–0.9) (Table 2). AIV were detected in seven taxa, including one sample from cattle egret feces and eight other samples from captured passerines (Table 1 and Additional fila 4). The resulting intragroup prevalence values ranged from 0.35% (95% CI: 0–2) in the *Muscicapidae* family to 3.0% (95% CI: 1–15) in the *Anthus* genus (Table 2). The Ct values ranged from 33.0 to 37.0 (mean of 34.5). Sequencing and identification of the HA gene from samples of captured passerines were attempted with no success, and all the results were negative for H6 real-time RT-PCR (Additional file 4). The positive cattle egret faeces presented a 1085 bp H5 sequence with a low pathogenic pattern (no polybasic sequence at the cleavage site), with the highest identity of 98.14% with strain A/mallard/Saskatchewan/17/1981(H5N2) (accession number CY178647) in the GenBank database.

**Table 2:** **Overall positive rate for each group of species sampled**

Group of species	Sample size PCR (birds or flocks)	PCR positive birds or flocks				Positive rate PCR (Wilson 95% CI)				Sample size ELISA (birds)	ELISA positive birds		Positive rate ELISA (Wilson 95% CI)	
		AIV	Avu	CoV	Chlam	AIV	Avu	CoV	Chlam		AIV	Avu	AIV	Avu
MUS	289	1	0	0	0	0.35 (0–2)	0.00 (0–1)	0.00 (0–1)	0.00 (0–1)	159	5	0	3.14 (1–7)	0.00 (0–2)
BUB	206	1	0	0	0	0.49 (0–3)	0.00 (0–2)	0.00 (0–2)	0.00 (0–2)	1	0	0	0 (0–79)	0 (0–79)
PASM	206	0	0	0	1	0.00 (0–2)	0.00 (0–2)	0.00 (0–2)	0.49 (0–3)	156	2	0	1.28 (0–5) **AB**	0.00 (0–2)
PASD	199	2	0	0	0	1.01 (0–4)	0.00 (0–2)	0.00 (0–2)	0.00 (0–2)	173	1	0	0.58 (0–3) **A**	0.00 (0–2)
FRI	186	1	0	0	1	0.54 (0–3)	0.00 (0–2)	0.00 (0–2)	0.54 (0–3)	45	0	0	0.0 (0–8)	0.0 (0–8)
SYL	169	2	0	0	2	1.18 (0–4)	0.00 (0–2)	0.00 (0–2)	1.18 (0–4)	76	5	0	6.6 (3–14) **BC**	0.0 (0–5)
AEG	142	0	0	0	3	0.00 (0–3)	0.00 (0–3)	0.00 (0–3)	2.11 (1–6)	0	–	–	–	–
PAR	133	0	0	0	0	0.00 (0–3)	0.00 (0–3)	0.00 (0–3)	0.00 (0–3)	37	0	0	0.0 (0–9)	0.0 (0–9)
TUR	117	0	0	0	0	0.00 (0–3)	0.00 (0–3)	0.00 (0–3)	0.00 (0–3)	100	3	2	3.0 (1–8)	2.00 (1–7)
MOT	91	1	0	0	2	1.1 (0–6)	0.0 (0–4)	0.0 (0–4)	2.2 (1–8)	58	5	1	8.6 (4–19)** C**	1.7 (0–9)
CER	37	0	0	0	0	0.0 (0–9)	0.0 (0–9)	0.0 (0–9)	0.0 (0–9)	7	0	0	0 (0–35)	0 (0–35)
PRU	35	0	0	0	0	0.0 (0–10)	0.0 (0–10)	0.0 (0–10)	0.0 (0–10)	30	3	0	10.0 (3–26) **C**	0.0 (0–11)
ANT	33	1	0	0	0	3.0 (1–15)	0.0 (0–10)	0.0 (0–10)	0.0 (0–10)	22	0	1	0.0 (0–15)	4.5 (1–22)
PIC	21	0	0	0	0	0.0 (0–15)	0.0 (0–15)	0.0 (0–15)	0.0 (0–15)	12	0	0	0 (0–24)	0 (0–24)
EMB	19	0	0	0	0	0 (0–17)	0 (0–17)	0 (0–17)	0 (0–17)	9	0	0	0 (0–30)	0 (0–30)
STU	19	0	0	0	0	0 (0–17)	0 (0–17)	0 (0–17)	0 (0–17)	11	0	0	0 (0–26)	0 (0–26)
REG	9	0	0	0	0	0 (0–30)	0 (0–30)	0 (0–30)	0 (0–30)	0	–	–	–	–
COR	6	0	0	0	0	0 (0–39)	0 (0–39)	0 (0–39)	0 (0–39)	3	0	0	0 (0–56)	0 (0–56)
ACC	4	0	0	0	0	0 (0–49)	0 (0–49)	0 (0–49)	0 (0–49)	4	1	0	25 (5–70) **BC**	0 (0–49)
CHA	4	0	0	0	0	0 (0–49)	0 (0–49)	0 (0–49)	0 (0–49)	3	0	0	0 (0–56)	0 (0–56)
ALC	3	0	0	0	0	0 (0–56)	0 (0–56)	0 (0–56)	0 (0–56)	1	0	0	0 (0–79)	0 (0–79)
HIR	3	0	0	0	0	0 (0–56)	0 (0–56)	0 (0–56)	0 (0–56)	1	0	0	0 (0–79)	0 (0–79)
STR	2	0	0	0	0	0 (0–66)	0 (0–66)	0 (0–66)	0 (0–66)	0	–	–	–	–
GAL	1	0	0	0	0	0 (0–79)	0 (0–79)	0 (0–79)	0 (0–79)	0	–	–	–	–
STRI	1	0	0	0	0	0 (0–79)	0 (0–79)	0 (0–79)	0 (0–79)	1	0	0	0 (0–79)	0 (0–79)
UPU	1	0	0	0	0	0 (0–79)	0 (0–79)	0 (0–79)	0 (0–79)	1	0	0	0 (0–79)	0 (0–79)
Ducks	5 (12 VIA)	5	0	2	2	42 (19–68)	0 (0–24)	40 (12–77)	40 (12–77)	–	–		–	–
Environment	16	6	0	3	7	38 (18–61)	0 (0–19)	19 (7–43)	44 (23–67)	–	–		–	–

The groups are as defined in Table 2. The Wilson 95% confidence interval (CI) is given between brackets. Duck positivity is given at the flock level. The sample size of duck flocks is larger for AIV screening because of regulatory testing, as specified between brackets. Bold letters after some values indicate groups of significantly different values (i.e., distinct letters) according to Chi-square tests (significance threshold is *p* = 0.05), with no letter indicating statistical similarity to all other groups. Groups of wild birds are coded as detailed in Additional file 1. AIV stands for avian influenza virus, Avu for avulaviruses, CoV for coronaviruses and Chlam for *Chlamydia* sp.

ELISA tests of 910 captured passerines detected antibodies in 24 individuals of 8 different taxa (2.75% corrected for Se and Sp, 95% CI: 1.9–4.0), with one *Passer montanus* positive in two consecutive months (Table 1 and Additional file 4). The resulting seroprevalence values ranged from 0.58% (95% CI: 0–3) in *Passer domesticus* to 25% in *Accipiter nisus* (95% CI: 5–70) (Table 2). Antibody levels resulted in competition percentages between 9.4% and 49.2% (mean of 32%), and none of the passerines were positive for H5-specific antibodies.

**Figure 1:**
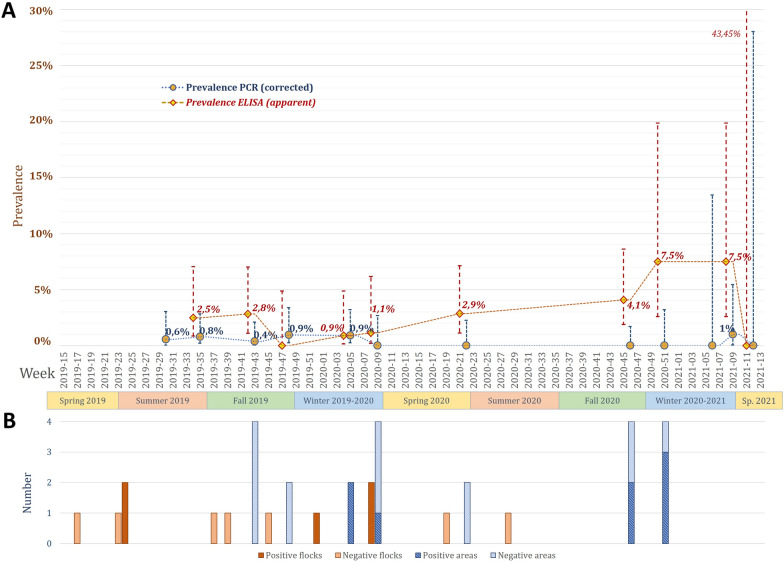
**Longitudinal screening of avian influenza virus detection in wild birds (A) and duck flocks and their areas (B)**. The PCR prevalence is shown as the corrected prevalence with test characteristics, and the ELISA prevalence is shown as the apparent prevalence. Error bars indicate 95% confidence intervals of prevalence.

Considering all the wild bird species detected over time, the apparent prevalence of AIV in swabs and faeces reached a maximum of 1.0% (95% CI: 0.17–5.40) in February 2021, which corresponded to corrected prevalence values of up to a maximum of 1.0% (95% CI: 0.05–5.45) (Figure 1). The seroprevalence of AIV reached a maximum of 7.5% (95% CI: 4.92–12.36) in both December 2020 and February 2021 (Figure 1).

#### Avulaviruses and coronaviruses in wild birds

None of the samples from the 1938 wild birds were positive for avulaviruses or coronaviruses according to real-time RT-PCR (Tables 1 and 2). The absence of detection by real-time RT-PCR corresponded to a positive rate, with an overall 95% CI of 0.0–0.2% (Table 2).

**Figure 2:**
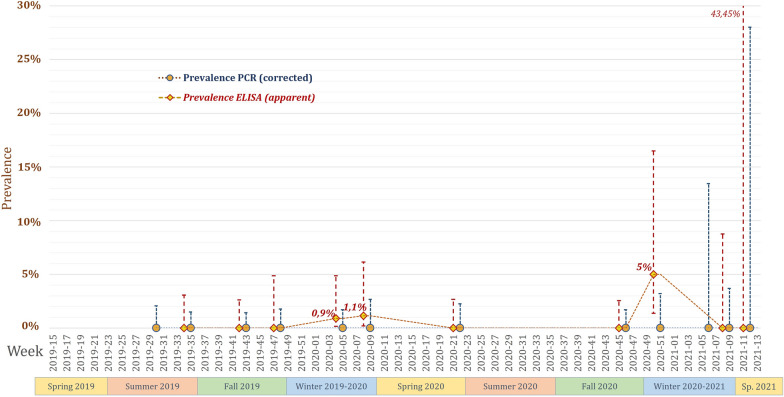
**Longitudinal screening of avulavirus and NDV detection in wild birds (duck flocks and areas were all negative)**. The PCR prevalence is shown as the corrected prevalence with test characteristics, and the ELISA prevalence is shown as the apparent prevalence. Error bars indicate 95% confidence intervals of prevalence.

Among the 910 blood samples, 4 were seropositive according to the NDV ELISA (0.44% corrected, 95% CI: 0.2–1.1), with competition percentages between 41.4% and 69.6% (mean of 58%) (Additional file 4). Two samples were from the same individual of *Turdus merula* one month apart, and the two others were one from *Anthus pratensis* and one from *Motacilla alba* (Table 1 and Additional file 4). These results revealed seroprevalence values between 1.7% (95% CI: 0–9) in the *Motacilla* genus and 4.5% (95% CI: 1–22) in the *Anthus* genus (Table 2). The prevalence of NDV among all wild birds reached a maximum of 5% (95% CI: 1.38–16.5) in December 2020 (Figure 2).

#### *Chlamydia* sp. in wild birds

Nine wild birds in 1938 were positive for *Chlamydia* sp. by real-time PCR (0.46% corrected, 95% CI: 0.2–0.9) (Tables 1 and 2). The positive samples were cloacal or oropharyngeal swabs from five different bird groups (Table 1 and Additional file 4). In these five groups, the prevalence values ranged from 0.49% (95% CI: 0–3) in *Passer montanus* to 2.2% (95% CI: 1–8) in the *Motacilla* genus (Table 2). The Ct values ranged from 27 to 40 (mean of 36) (Additional file 4). MLST was successful on samples from three birds, which were shown to carry two different strains of avian *Chlamydia abortus* corresponding to a new MLST sequence type: one in two individuals of *Phylloscopus collybita* (ST329) and another in *Sylvia communis* (ST330) (Additional file 5). The presence of the specific plasmid of these avian strains was confirmed for each of these samples (Additional file 5). The cloacal swab from a *Fringilla coeleb* was positive for the specific real-time PCR for *C. psittaci*, but further identification was unsuccessful (Additional file 4). The five other positive wild bird samples presented the highest Ct values with real-time PCR for *Chlamydia* sp.; these samples were negative with specific real-time PCR for *C. psittaci* or *C. abortus* and were not successfully identified (Additional file 4).

Considering all the wild bird species present over time, the apparent prevalence values of *Chlamydia* sp. in swabs and faeces reached a maximum of 1.4% (95% CI: 0.63–7.91) in February 2020, which corresponded to corrected prevalence values of up to a maximum of 2.3% (95% CI: 0.63–7.99) (Figure 4).

#### Comparison of pathogens in wild birds

Overall, the wild birds that tested positive by PCR or ELISA for any of the pathogens belonged to 12 of the 26 groups in total. In all 12 groups, only the *Motacilla* genus was positive for both AIV (1.1%, 95% CI: 0–6), *Chlamydia* sp. (2.2%, 95% CI: 1–8), AIV (8.6%, 95% CI: 4–19) and NDV (1.7%, 95% CI: 0–9) antibodies (Table 2). Chi-square tests were performed on the PCR prevalence values of the wild bird groups, and the results revealed no significant differences (*p* values over 0.6 for AIV and *Chlamydia* sp.). The seroprevalence values of NDV were statistically similar between the groups, but slightly significant differences were detected for AIV (X^2^ = 33.7, *p* = 0.038) (Table 2). The seroprevalence of *Passer domesticus* was significantly lower (0.6%, 95% CI: 0–3) than that of *Accipiter nisus* (25%, 95% CI: 5–70, X^2^ = 4.7, *p* = 2.9e-2), *Prunella modularis* (10.0%, 95% CI: 3–26, X^2^ = 7.4, *p* = 6.6e-3), the *Motacilla* genus (8.6%, 95% CI: 4–19, X^2^ = 8.1, *p* = 4.3e-3), and the *Sylviida* parvorder (6.6%, 95% CI: 3–14, X^2^ = 5.7, *p* = 1.7e-2). The seroprevalence of *Passer montanus* was significantly lower (1.3%, 95% CI: 0–5) than that of *Prunella modularis* (10.0%, 95% CI: 3–26, X^2^ = 4.4, *p* = 3.7e-2) and the *Motacilla* genus (8.6%, 95% CI: 4–19, X^2^ = 5.1, *p* = 2.4e-2).

### Detection and identification of pathogens in domestic ducks

#### AIV in domestic ducks

Five of the 12 flocks were positive for the M gene according to real-time RT-PCR (Table 2, Figure 1). Three strains were detected from swabs for regulatory surveillance: two in the same week, identified by the NRL as H7N3 (low pathogenic), and another negative for H5 or H7 subtypes. The other two positive flocks (cloacal swabs) were negative for H5 or H7 subtypes. The Ct values from duck swabs were lower than those obtained from wild birds, ranging from 30.7 to 34.3 (mean Ct of 32.9). The H6 subtype was identified by sequencing in these two flocks (Additional file 4), and both sequences were similar. The closest H6 sequence in the GenBank database was from the strain A/mallard duck/Georgia/7/2015 (H6N1) (accession number MF694213) isolated in central Eurasia, with over 98% identity at 610–670 bp. One positive swab from one of these two flocks also resulted in a different HA sequence (554 bp and poor quality) identified as H11 with 91.3% identity to strain A/Anas platyrhynchos/Belgium/195_7/2018 (H11N9) (accession number MT406953) from northwestern Europe.

#### Avulaviruses in domestic ducks

None of the swabs from the five duck flocks analysed were positive for avulaviruses according to real-time RT-PCR (Tables 1 and 2).

#### Coronaviruses in domestic ducks

**Figure 3:**
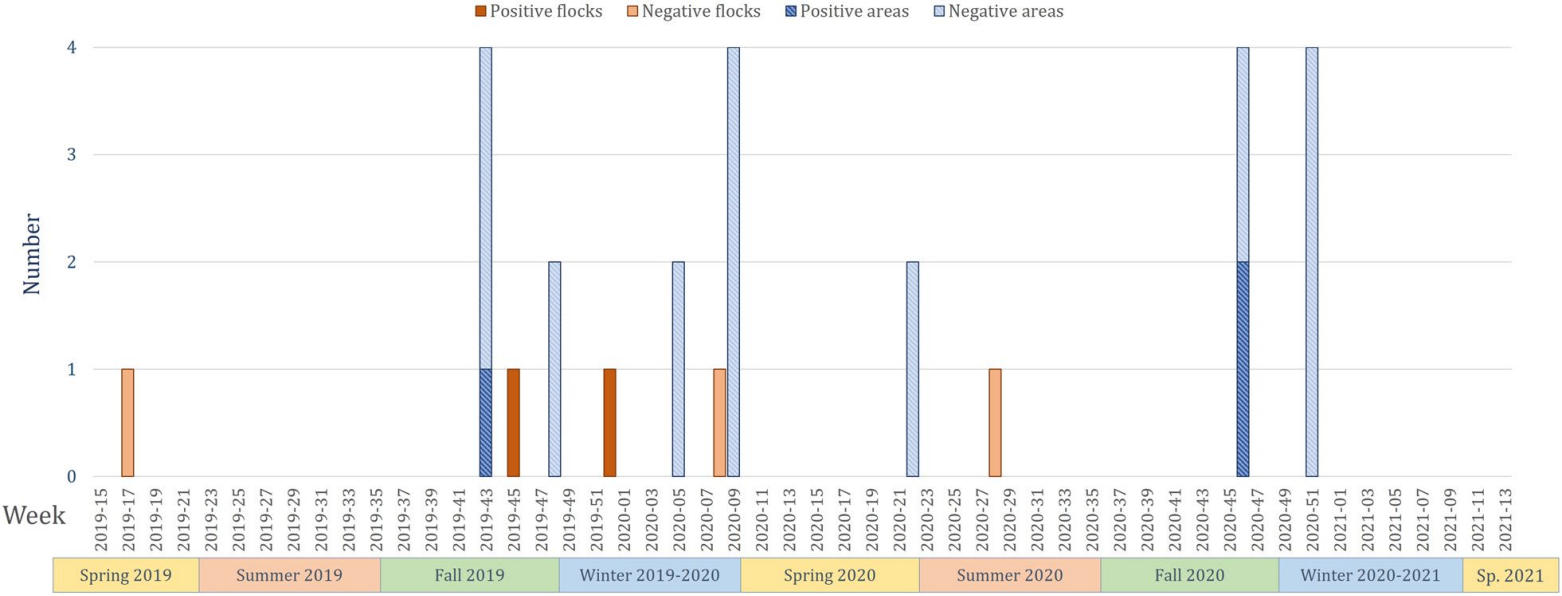
**Longitudinal screening of coronavirus detection in duck flocks and areas (wild birds were all negative).**

Two of the five duck flocks were positive for coronaviruses according to real-time RT-PCR (Tables 1, 2 and Figure 3), both from cloacal swabs. The Ct values of these positive samples ranged from 31.7 to 39.2 (mean of 35.7). All swabs were successfully sequenced on 550–600 bp of the coronavirus polymerase gene, and all were similar and identified as duck coronavirus 2714 (gammacoronavirus), with 94–95% identity to strains from wild Anatidae (for example, GenBank accession number MK204411) and the duck coronavirus isolate DK/GD/27/2014 (accession number NC_048214).

#### *Chlamydia* sp. in domestic ducks

**Figure 4:**
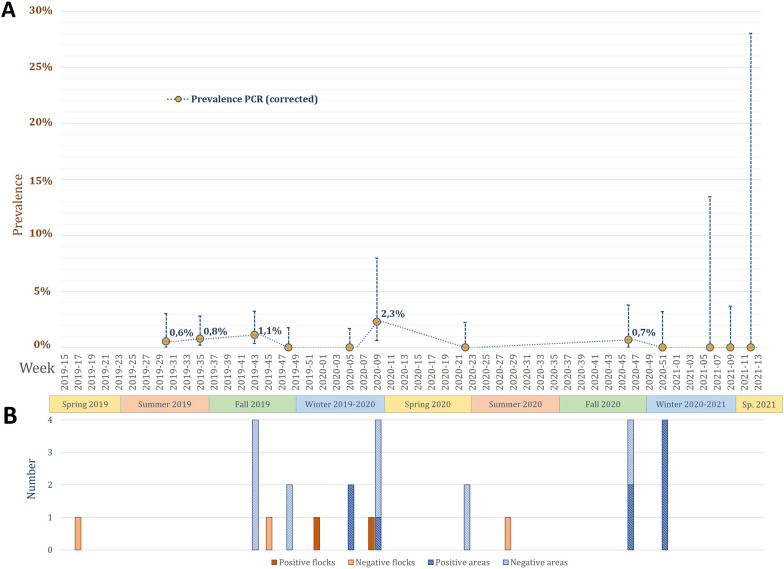
**Longitudinal screening of** ***Chlamydia* sp. detection in wild birds (A) and duck flocks and their areas** **(B) over time**. The PCR prevalence is shown as the corrected prevalence with test characteristics. Error bars indicate 95% confidence intervals of prevalence.

Two of the five duck flocks were positive for *Chlamydia* sp. according to real-time PCR (Tables 1, 2 and Figure 4): oropharyngeal and cloacal swabs from one flock and only cloacal swabs from the other flock. The Ct values ranged from 26.5 to 37.5 (mean of 33.8). All positive samples were successfully identified as *C. psittaci* strains via real-time PCR and classified into Group II_Duck via PCR-HRM (data not shown).

#### Comparison of pathogens in domestic ducks

Positivity for pathogens in duck swabs varied over time, from no detection (in April 2019 and July 2020) to simultaneous detection of AIV, coronaviruses and *Chlamydia* sp. in the same flock (in December 2019). Except for the two AIV-positive flocks detected in the summer of 2019, each of the three agents were detected only in ducks during the fall or winter seasons (Figures 1, 3, 4). However, owing to the low number of flocks, chi-square tests calculated on prevalence values over time in the duck flocks revealed no significant differences (*p* values over 0.2).

### Detection and identification of pathogens in the environment of the farm

#### AIV in the environment

In the environment of the duck outdoor areas, 6 out of 16 areas were positive for the M gene according to real-time RT-PCR (Tables 1, 2 and Figure 1). The Ct values ranged from 31.0 to 35.2 (mean Ct of 33.3). The H6 subtype was identified either by sequencing or specific real-time RT-PCR in all but one positive area (Additional file 4). The sequence from H6 AIV was similar to that obtained from duck swabs.

#### Avulaviruses in the environment

None of the environmental samples were positive for avulaviruses according to real-time RT-PCR (Tables 1 and 2).

#### Coronaviruses in the environment

Three of the 16 duck areas were positive for coronaviruses according to real-time RT-PCR (Tables 1, 2 and Figure 3). The Ct values of these samples ranged from 33.3 to 40.0 (mean of 37.3). The environmental sample from October 2019 was successfully sequenced on 550 bp of the coronavirus polymerase gene and was similar to sequences from duck swabs.

#### *Chlamydia* sp. in the environment

Six of the 16 duck areas were positive for *Chlamydia* sp. according to real-time PCR (Table 1, Table 2 and Figure 3). Two of them were positive in the same two consecutive months (Additional file 4). The Ct values of the environmental samples ranged from 29.7–36.6 (mean of 34.0). The positive environmental samples from January, February and December 2020 were identified as the same *C. psittaci* strains detected in the duck swabs (SNP Group II_Duck) (Additional file 4). In addition, *C. abortus* strains were detected in three areas sampled from November–December 2020, one of which was also positive for *C. psittaci* (Additional file 4). Unfortunately, the low bacterial load of these samples prevented their identification.

#### Comparison of pathogens in the environment

Positivity for pathogens in the environment of duck areas has varied over time, from no detection (in November 2019 and May 2020) to detection of AIV, coronaviruses and *Chlamydia* sp. at the same time in the same area (in November 2020). Each of the three agents was detected only in the environment during the fall or winter seasons (Figures 1, 3 and 4). However, owing to the low number of areas, chi-square tests calculated on prevalence values over time revealed few significant differences (most *p* values were greater than 0.05). Only the prevalence of *Chlamydia* sp. was significantly greater in December 2020 (four areas positive out of four) than in October 2019 (four areas negative out of four) (X^2^ = 4.5, *p* = 3.4e-2).

### Comparison of avian pathogens in the three compartments

**Figure 5:**
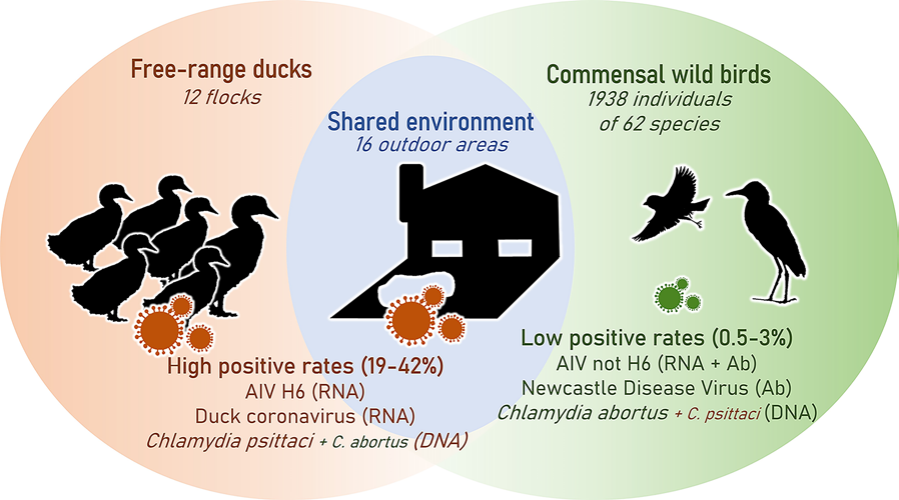
**Global comparison of avian pathogens detected in the three compartments of the interface.** Ranges of positive rates are given between brackets for detected pathogens only. Ab: antibodies.

For AIV, duck swabs were positive for 5 flocks out of 12 and environmental samples for 6 areas out of 16, with 2 flocks showing positive results in their swabs conjointly with the environment of their area (Table 1). Overall, ducks and the environment presented statistically similar rates of AIV positivity according to chi-square tests (X^2^ = 0, *p* = 1). However, both ducks and the environment presented much higher viral positive rates than did any other wild bird taxa (X^2^ = 419.9, *p* < 1e-10) (Figure 5).

For coronaviruses, duck swabs were positive for 2 flocks out of 5 and environmental samples for 3 areas out of 16, but no duck flock was positive conjointly with environmental samples from their area (Table 1). Overall, the rates of coronavirus positivity in ducks and the environment were statistically similar according to chi-square tests (X^2^ = 0.1, *p* = 0.7). As no wild bird was detected as positive by real-time RT‒PCR, both ducks and the environment presented significantly higher positive rates than did the wild bird taxa (X^2^ = 530.2, *p* < 1e-10) (Figure 5).

For *Chlamydia* sp., duck swabs were positive for 2 flocks out of 5 and environmental samples for 7 areas out of 16, with one duck flock showing positive results in swabs conjoint with environmental samples of their area (Table 1). Overall, ducks and the environment presented statistically similar rates of *Chlamydia* sp. bacterial positivity according to chi-square tests (X^2^ = 0, *p* = 1). However, both ducks and the environment presented much higher bacterial positive rates than any other wild bird taxa did (X^2^ = 421.6, *p* < 1e-10) (Figure 5).

## Discussion

The present study highlights the epidemiology of major pathogens of the least studied population and context, namely, commensal birds around a free-range duck farm in Europe. The prevalence levels of AIV in domestic ducks and wild birds observed in the present study were globally consistent with published results on similar (i.e., terrestrial commensal) species in a non-epidemic context. AIV have often been detected by molecular tests at very low or even null prevalence levels in passerines and other terrestrial birds [15, 18, 46], whereas antibodies have often shown slightly higher prevalence levels [18, 38, 46]. The almost completely negative prevalences found for NDV and avulaviruses were also expected, since commercial poultry are free of NDV in France [47], and the Passeriformes sampled in this study are not commonly found to be positive in most contexts in comparison with, for instance, waterfowl and Columbiformes [16, 18, 48]. The gamma genus of coronaviruses is widely distributed in waterfowl (mostly Anseriformes and Charadriiformes) with various strains [20], which correlates with the results observed in the duck flocks in this study. In contrast, Passeriformes are generally the least infected, and when they are, delta genus strains are usually involved [20]. Finally, the results of *Chlamydia* sp. detection were consistent with those of previous studies in free-range ducks from France that were frequently infected by *C. psittaci* in flocks with high intra-flock prevalence [23]. In wild birds, *C. psittaci* are more common in waterfowl or Columbiformes than in passerines, as was observed in the present study [21, 27]. Avian strains of *C. abortus,* which have just been recently described in seabirds and corvids [25–27] and for which detection tools are now available [25, 26], were also detected in avian wildlife in this study, for the first time in France.

The present study also contributes to original knowledge on the fine-scale longitudinal epidemiology of avian pathogens, helping to better understand infection dynamics in the populations studied. Only a handful of other studies have implemented comparable methods, for instance, in domestic duck flocks for *Chlamydia* sp. [23, 49], in wild swans for AIV [50], and in various peridomestic birds for NDV [40]. The temporal patterns of AIV in wild birds and duck flocks identified in the present study did not significantly differ. This might be due to the relatively small size of the samples involved each time, in combination with the low positivity rate. However, the evaluation of population sensitivity carried out for each wild bird sampling session provided good confidence that no epidemic trend was missed, with a detectable threshold of population prevalence generally below 3% for molecular testing and 8% for serological testing (Additional file 6). It also appears that AIV circulation dynamics are not correlated between ducks and wild birds (Figure 1), even with serological testing, which allows longer detection times than does viral testing (a few weeks rather than a few days) in species of low susceptibility [51–53]. These non-matching patterns add to differences in the identification of AIV strains between wild and domestic birds. No AIV from wild birds can be identified precisely by isolation and sequencing, which is an ongoing issue in terrestrial wild bird studies due to the low quantities of biological material available from small and asymptomatic birds [15, 54]. However, at least the H6 subtype of AIV was excluded by specific real-time RT-PCR. As this subtype is the most frequently identified in ducks, its transmission to wild birds seems to be null or at least lower than estimated detection thresholds in wild birds (Additional file 6). Moreover, the similarity of H6 AIV sequences over two consecutive years, as well as those of duck coronavirus and *C. psittaci* from ducks or from their environment, suggests endemic circulation of these agents on farms, with limited intermediate introduction by wild birds or other pathways. Owing to the low probability of infectious transmission at the interface and the low prevalence detected in the commensal birds sampled on the studied duck farm, the role of commensal wild birds as maintenance hosts for AIV (and probably for the three other avian pathogens) seems to be excluded, as evidenced previously [15]. However, sporadic transmission of AIV between wild birds and poultry still cannot be excluded, especially in cases of the emergence of highly pathogenic strains that could be excreted more abundantly by these hosts in a similar context [13, 53]. In such contexts of HPAI circulation, sporadic transmissions could be sufficient for mobile commensal birds to spread viruses from one farm to another or to any other susceptible population by individual movements during the few days of excretion. The findings of the present study suggest that the conceptual role of bridge hosts for AIV [14] might be played by a few commensal wild bird species on rare occasions.

The results of this study provide evidence of the technical difficulties associated with characterizing AIV circulation in terrestrial birds. When captures that target asymptomatic individuals of mostly small species are used, very low prevalence rates and very low viral loads in samples are expected, which makes direct detection and identification from swabs difficult even around acute farm outbreaks [54–57]. To improve the sensitivity of AIV, alternative and complementary methods, such as serological testing in wild birds and sampling of various materials of the triad of domestic ducks, wild birds and their shared environment, were used in this study. Moreover, to characterize AIV transmission pathways, the present study targeted three other avian pathogens as infectious transmission markers. To our knowledge, the simultaneous study of other pathogens as transmission markers for AIV is innovative at the wild-Chinese interface. In this study, strain identification was more successful and complete for *Chlamydia* sp. than for AIV, which suggests that this bacterial taxon is a good choice as a transmission marker at the studied wild-domestic interface. Successful strain identification for AIV and *Chlamydia* sp. was always inconsistent between the two compartments (ducks and commensal birds). Indeed, only *Chlamydia psittaci* strains belonging to the SNP Group II_Duck were identified in duck swabs, whereas mostly avian *C. abortus* strains were identified in commensal birds. Moreover, the sequences of H6 AIV, as well as those of duck coronavirus and *C. psittaci* retrieved from samples of ducks or from their environment, were similar over two consecutive years. The evidence of such similarity in other pathogens in parallel with AIV strengthens the hypothesis of an endemic circulation of pathogens on farms and strengthens the interest in studying infectious markers to reveal the epidemiological patterns of AIV.

In addition to considering several pathogens and using several sample materials, an original feature of the present study is its focus on the three compartments of the epidemiological interface, namely, domestic ducks, wild birds and their shared environment, of a single farm. To our knowledge, this triad has never been studied in a real-life setting; however, it has been studied under experimental conditions [52, 53]. Environmental samples of surface water and wipes were collected from farms in outdoor areas where ducks are present. The nature of these samples seemed to provide a good picture of infectious shedding at the scale of each flock, as molecular detection and identification are usually similar in ducks (swabs) and their environment. As duck flocks represent a concentration of thousands of large, young birds that live for several weeks in these areas, high loads of duck infectious agents can be maintained and consequently are widely dominant locally in comparison with wild bird infectious agents, as was observed in this study. This microbiological contamination of the environment would very likely expose local avian communities to the pathogens of ducks, and the reverse way seems much less important under these conditions.

Although general contamination of duck flocks and their outdoor environment at the same time (for instance, with AIV and *Chlamydia* sp. during the winters of 2019–2020 and fall 2020) did not have an important effect on the infection of commensal wild birds repeatedly observed in contact with these two compartments [10]. This impact was at least not visible in most asymptomatic birds captured in this study, and sick or dead birds were never observed at the study site. However, notably, the seroprevalence of AIV in wagtails (mostly White wagtail, *Motacilla alba*) was among the highest of all the wild bird groups, and it was significantly greater than that in both sparrow species (*Passer domesticus* and *P. montanus*). As these three species were previously shown to be the most abundant in contact with domestic ducks and their environment on farms [10], this difference in AIV seroprevalence may imply a difference in transmission risk associated with such contacts. Although AIV strains could not be identified in wild birds in this study to support possible transmission at the interface, the intense contact of white wagtails with the farm and their mobile ecology may favour spillover and spillback of AIVs between free-range ducks and other avian communities. These results suggest that white wagtails may play a role as potential bridge hosts, at least for AIV, between poultry farms and wetlands in southwestern France, and this role might exist in other regions where the species is present on farms [58]. Nevertheless, the seroprevalence in wagtails was globally low (8.6%) compared with the high intensity of contact they had with duck flocks, which occasionally shed AIVs in contaminated environments. The differences in positive rates and strain identifications despite intense mutual contacts support the idea of a general species barrier at the interface between domestic ducks and commensal wild birds. This has also been observed for bacteria of the genus *Mycoplasma* in the same region of southwestern France [59]. However, in the context of the circulation of a highly pathogenic agent with a broader host range, such as HPAIV, the epidemiological role of terrestrial commensal wild birds on farms may be intensified. Nevertheless, the repeated rare to null detection of HPAIV in terrestrial wild birds around farm outbreaks [55, 56, 60, 61] supports their very sporadic role in viral spread between and to farms, a role that is generally overwhelmed by other pathways in transmission dynamics on a regional scale [9, 62].

## Supplementary Information

**Additional file 1. Description of sampled wild birds by taxonomic group.**

**Additional file 2. Protocols for preparation of samples and nucleic acid extraction.**

**Additional file 3. Optimization processes and final protocols for the molecular detection of avian influenza viruses, avulaviruses, coronaviruses and** ***Chlamydia* sp.**

**Additional file 4. Description of positive samples for all four infectious agents.**

**Additional file 5. Phylogeny of the identified** ***Chlamydia* sp.**

**Additional file 6. Population sensitivity for each time of wild bird sampling.**

## Data Availability

The datasets used and analysed during the current study are available from the corresponding author upon reasonable request.
