# Supplementary material for: Limited transmission of avian influenza viruses, avulaviruses, coronaviruses and Chlamydia sp. at the interface between wild birds and a free-range duck farm
Source: Vet Res. 2025 Feb 8;56:36. doi: 10.1186/s13567-025-01466-3 (PMC11806813; doi:10.1186/s13567-025-01466-3)
Supplement: Supplementary file 1 — Additional file 1. Description of sampled wild birds by taxonomic group. [file 13567_2025_1466_MOESM1_ESM.docx]

*Description of sampled wild birds by taxonomic group*

**Supplementary Table 1.** Description of sampled wild birds by taxonomic group.
Passeriformes groups are established from Oliveros et al., 2019. All samples are from captured birds, except feces from roosting cattle egrets (*Bubulcus ibis*) as specified by “(+ 207 feces)” on the corresponding line.

| **Order** | **Family** | **Group code** | **Species in the group (by decreasing numbers)** | **Number of swabs** | **Number of sera** |
| --- | --- | --- | --- | --- | --- |
| Passeriformes | Sylviidae; Acrocephalidae; Alaudidae | **SYL** | Eurasian Blackcap *Sylvia atricapilla* (SYLATR)*;* Garden warbler *Sylvia borin;* Common Whitethroat *Sylvia communis;* Common Reed Warbler *Acrocephalus scirpaceus;* Melodious Warbler *Hippolais polyglotta;* Woodlark *Lullula arborea* | **169** | **76** |
|  | Aegithalidae; Phylloscopidae | **AEG** | Common Chiffchaff *Phylloscopus collybita* (PHYCOL)*;* Long-tailed Tit *Aegithalos caudatus;* Willow Warbler *Phylloscopus trochilus* | **142** | **0** |
|  | Certhiidae; Sittidae; Troglodytidae | **CER** | Short-toed Treecreeper *Certhia brachydactyla;* Eurasian Nuthatch *Sitta europaea;* Eurasian Wren *Troglodytes troglodytes* | **37** | **7** |
|  | Corvidae; Oriolidae | **COR** | Eurasian Jay *Garrulus glandarius;* Eurasian Golden Oriole *Oriolus oriolus;* Eurasian Magpie *Pica pica* | **6** | **3** |
|  | Emberizidae | **EMB** | Cirl Bunting *Emberiza cirlus;* Common Reed Bunting *Emberiza schoeniclus* | **19** | **9** |
|  | Fringillidae | **FRI** | Eurasian Chaffinch *Fringilla coelebs;* European Goldfinch *Carduelis carduelis;* European Greenfinch *Chloris chloris;* Hawfinch *Coccothraustes coccothraustes;* Brambling *Fringilla montifringilla;* Common Linnet *Linaria cannabina;* European Serin *Serinus serinus* | **186** | **45** |
|  | Hirundinidae | **HIR** | Barn Swallow *Hirundo rustica;* Western House Martin *Delichon urbicum* | **3** | **1** |
|  | Motacillidae | **ANT** | Meadow Pipit *Anthus pratensis* (ANTPRA)*;* Water Pipit *Anthus spinoletta;* Tree Pipit *Anthus trivialis* | **33** | **22** |
|  |  | **MOT** | White Wagtail *Motacilla alba* (MOTALB)*;* Grey Wagtail *Motacilla cinerea* | **91** | **58** |
|  | Muscicapidae | **MUS** | European Robin *Erithacus rubecula* (ERIRUB)*;* European Pied Flycatcher *Ficedula hypoleuca* (FICUCA)*;* Black Redstart *Phoenicurus ochruros;* Common Nightinglae *Luscinia megarhynchos* (LUSMEG)*;* Common Redstart *Phoenicurus phoenicurus;* European Stonechat *Saxicola rubicola;* Spotted Flycatcher *Muscicapa striata* | **289** | **159** |
|  | Paridae | **PAR** | Eurasian Blue Tit *Cyanistes caeruleus;* Great Tit *Parus major;* Coal Tit *Periparus ater* | **133** | **37** |
|  | Passeridae | **PASD** | House Sparrow *Passer domesticus* | **199** | **173** |
|  |  | **PASM** | Eurasian Tree Sparrow *Passer montanus* | **206** | **156** |
|  | Prunellidae | **PRU** | Dunnock *Prunella modularis* | **35** | **30** |
|  | Regulidae | **REG** | Common Firecrest *Regulus ignicapilla;* Goldcrest *Regulus regulus* | **9** | **0** |
|  | Sturnidae | **STU** | Common Starling *Sturnus vulgaris* | **19** | **11** |
|  | Turdidae | **TUR** | Common Blackbird *Turdus merula* (TURMER)*;* Song Thrush *Turdus philomelos* (TURPHI)*;* Redwing *Turdus iliacus* | **117** | **100** |
| Pelecaniformes | Ardeidae | **BUB** | Cattle Egret *Bubulcus ibis* | **1 (+ 207 feces)** | **1** |
| Accipitriformes | Accipitridae | **ACC** | Eurasian Sparrowhawk *Accipiter nisus* | **4** | **4** |
| Bucerotiformes | Upupidae | **UPU** | Eurasian Hoopoe *Upupa epops* | **1** | **1** |
| Charadriiformes | Scolopacidae | **CHA** | Common Snipe *Gallinago gallinago;* Yellow-legged Gull *Larus michahellis* | **4** | **3** |
| Columbiformes | Columbidae | **STR** | Eurasian Collared Dove *Streptopelia decaocto* | **2** | **0** |
| Coraciiformes | Alcedinidae | **ALC** | Common Kingfisher *Alcedo atthis* | **3** | **1** |
| Galliformes | Phasianidae | **GAL** | Common Pheasant *Phasianus colchicus​* | **1** | **0** |
| Piciformes | Picidae | **PIC** | European Green Woodpecker *Picus viridis;* Great Spotted Woodpecker *Dendrocopos major;* Eurasian Wryneck *Jynx torquilla* | **21** | **12** |
| Strigiformes | Strigidae | **STRI** | Long-eared Owl *Asio otus* | **1** | **1** |
