# Supplementary material for: Limited transmission of avian influenza viruses, avulaviruses, coronaviruses and Chlamydia sp. at the interface between wild birds and a free-range duck farm
Source: Vet Res. 2025 Feb 8;56:36. doi: 10.1186/s13567-025-01466-3 (PMC11806813; doi:10.1186/s13567-025-01466-3)
Supplement: Supplementary file 2 — Additional file 2. Protocols for preparation of samples and nucleic acid extraction. [file 13567_2025_1466_MOESM2_ESM.docx]

*Protocols for preparation of samples and nucleic acid extraction*

# Preparation of samples

## Tracheal and cloacal swabs

Swabs in 300 µL 1X PBS were vortexed for 5 seconds then the supernatant was used for nucleic acids extraction.

## Environmental samples

Wipes in PBS were shaken at 45 movements per min (mpm) overnight at 6°C to dissolve solid organic particles. They were then firmly squeezed to extract the most liquid possible. Water samples were gently centrifuged to separate larger soil particles at 300 Relative Centrifugal Force (RCF) for 2 min.

In order to separate extracellular DNA from particles of soil and organic matter that may inhibit PCR reactions, supernatant of water samples (50 mL), as well as 20 mL of the wipes’ supernatants, were mixed with saturated phosphate salts [1add]: NaH_2_PO_4_ and Na_2_HPO_4_ were added in each sample to 1.97 g/L and 14.7 g/L concentrations, respectively. Samples were then shaken at 65 mpm for 15-30 min at 6°C, then tubes were centrifuged at 2,000 RCF for 5 min at 4°C to separate particles from suspended genetic material. Whole volumes of supernatant were then filtered using individual sterile 0.22 µm PES vacuum filters (Corning, New York, USA). Each filter membrane was detached from its frame and cut into halves in sterile conditions to be folded into 2 mL multi-size ceramic bead tubes (CKMix50-R from Bertin Corp., Rockville, Maryland, USA) with 1 mL sterile 1X PBS. Bead tubes were vortexed with a Precellys®24 instrument (Bertin Corp., Rockville, Maryland, USA) for 3 sessions of 30 s at 5,500 mpm to grind filter membranes and separate genetic materials. They were then centrifuged for 1 min at 1,200 RCF and supernatant was used for nucleic acids extraction.

# Nucleic acid extractions

For extraction and subsequent screening analyses, swab supernatants were pooled by 150 µL of same host (species or family), sample type (oral, tracheal or cloacal), and period of sampling (5 samples in each pool whenever possible). Environmental samples were processed by pools of 150 µL of the same period and location (water and wipes of the same foraging area).

Both RNA and DNA extractions from environment or swabs were performed from 150 µL of supernatant, using the NucleoMag Pathogen kit (Macherey-Nagel, Dueren, Germany) on a KingFisher Flex robot (Thermo Fisher Scientific Inc., Waltham, Massachusetts, USA), and following the manufacturer’s instructions. Negative extraction controls were processed alongside each series of extractions.

1add. Taberlet P, Prud’Homme SM, Campione E, Roy J, Miquel C, Shehzad W, Gielly L, Rioux D, Choler P, Clément J-C, Melodelima C, Pompanon F, Coissac E (2012) Soil sampling and isolation of extracellular DNA from large amount of starting material suitable for metabarcoding studies. Mol Ecol 21:1816–1820. https:// doi. org/ 10. 1111/j. 1365-​294X. 2011. 05317.x
