# Supplementary material for: Limited transmission of avian influenza viruses, avulaviruses, coronaviruses and Chlamydia sp. at the interface between wild birds and a free-range duck farm
Source: Vet Res. 2025 Feb 8;56:36. doi: 10.1186/s13567-025-01466-3 (PMC11806813; doi:10.1186/s13567-025-01466-3)
Supplement: Supplementary file 3 — Additional file 3. Optimization processes and final protocols for the molecular detection of avian influenza viruses, avulaviruses, coronaviruses and Chlamydia sp. [file 13567_2025_1466_MOESM3_ESM.docx]

***Optimization processes and final protocols for molecular detection of avian influenza viruses, avulaviruses, coronaviruses and Chlamydia sp.***

# Design and optimization of molecular detection tests

To screen swabs, feces and environmental samples for avulaviruses, coronaviruses and *Chlamydia* sp., broad range one-step real-time RT-PCR and real-time PCR were adapted for primer pairs that were previously described in the literature. To be able to differentiate a wide diversity of strains, protocols were optimized for SYBR Green technology on a LightCycler 96 thermocycler (Roche, Basel, Switzerland).

## Avulaviruses

To screen for potentially unidentified avulaviruses, a one-step real-time RT-PCR was adapted from a previously described protocol with AVU-RUB-F1 (5’-GGT TAT CCT CAT TTI TTY GAR TGG ATH CA-3’) and AVU-RUB-R (5’-GCA ATT GCT TGA TTI TCI CCY TGN AC-3’) primers targeting 200bp of the L gene of *Avulavirinae* and *Rubulavirinae* subfamilies [34]. Reactions were processed on a LightCycler 96 thermocycler (Roche, Basel, Switzerland) by using the iTaq Universal SYBR green One-Step kit (Biorad, Hercules, CA, USA) following the manufacturer’s instructions with a few modifications described below. Two dilutions of extracted RNA of a cultivated and identified NDV class II virus from a clinical case available at the laboratory were tested, on different annealing temperatures at 45, 50, 55 or 60°C. The highest sensitivity was obtained with an annealing temperature of 55°C, which was then selected for further analyses. The reaction mix used for subsequent analyses then consisted of 2 µL of sample RNA, 0.6 µL of each 10 µM primer (final concentration 0.6 µM), 5 µL of 2X iTaq reaction mix, 0.125 µL of iScript reverse transcriptase, completed with 1.675 µL of PCR-grade water for a total reaction volume of 10 µL. The thermocycler program consisted of a first 10 min pre-incubation step at 50°C, followed by 1 min at 95°C, then 40 amplification cycles including one 95°C step for 15 s, one 55°C annealing step for 15 s, and one 60°C step for 1 min. A final melting analysis step was then added following the manufacturer’s instructions. As a quantification control, a plasmid construct was synthetized by GenScript Biotech (Leiden, the Netherlands) including the target L gene sequence from Avian avulavirus 1 isolate duck/U.S./119535-1/2001 retrieved on GenBank (accession number AY626266.1) and adapted for few nucleotides to match the primers’ sequences. The selected real-time RT-PCR protocol was tested on the previous NDV class II dilutions and on this plasmid on a dilution range from 10 to 10^5^ copies/µL to validate its sensitivity (**Supplementary Figure 1**), estimated around 10^2^ RNA copies/µL.

## Coronaviruses

To screen for potentially unidentified coronaviruses, a one-step real-time RT-PCR was adapted from a previously described protocol with AC-CoV-F (5’-GGT TGG GAT TAT CCW AAR TGT G-3’) and AC-CoV-R (5’-TGY TGT GAR CAA AAY TCR TG-3’) primers targeting 600bp of the polymerase gene of all coronaviruses [35]. Reactions were processed on the same machine with the same one-step real-time RT-PCR kit described for avulaviruses. A first optimization was conducted for annealing temperature on 1/10 dilutions of SARS-CoV-2 RNA (betacoronavirus) and vaccine H52 infectious bronchitis virus (IBV) RNA (gammacoronavirus). The highest sensitivity and least primer self-complementarity was obtained with an annealing temperature of 50°C, which was then selected for further analyses. The reaction mix used for subsequent analyses was the same as for avulaviruses. The thermocycler program was similar to the one for avulaviruses, with an annealing step at 50°C instead of 55°C. As a quantification control, a plasmid construct including the target sequence from SARS-CoV-2 was built with StrataClone PCR Cloning Kit (Agilent technologies, Inc., Santa Clara, CA, USA) following the manufacturer’s instructions. The selected real-time RT-PCR protocol was tested on two dilutions of the previous H52 IBV RNA and on this plasmid on a dilution range from 10 to 10^6^ copies/µL to validate its sensitivity (**Supplementary Figure 1**), estimated around 10 RNA copies/µL.

## *Chlamydia* sp.

A previously described protocol for pan-chlamydia real-time PCR using Ch23S-F (5'-CTG AAA CCA GTA GCT TAT AAG CGG T-3') and Ch23S-R (5'-ACC TCG CCG TTT AAC TTA ACT CC-3') primers targeting a portion of the 23S rDNA [36] was adapted for SYBR Green technology. Reactions were processed on the same machine as described above, by using LightCycler SYBR Green I Master reaction mix (Roche, Basel, Switzerland) and following the manufacturer’s instructions. The reaction mix consisted of 2 µL of extracted DNA solution, with 0.4 µL of each 10 µM primer (final concentration of 0.2 µM), 10 µL of SYBR Green I Master, and completed with 7.2 µL of PCR-grade water for a total reaction volume of 20 µL. The thermocycler program consisted of a first 5 min pre-incubation step at 95°C, followed by 45 amplification cycles including 10 s at 95°C, 15 s at 55°C and 15 s at 72°C. A final melting analysis step was then added following the manufacturer’s instructions. As a quantification control, a plasmid construct including the target sequence from a cultivated *Chlamydia psittaci* was built with the StrataClone PCR Cloning Kit (Agilent technologies, Inc., Santa Clara, CA, USA) following the manufacturer’s instructions. The selected real-time PCR protocol was tested on this plasmid on a dilution range from 10 to 10^6^ copies/µL to validate its sensitivity (**Supplementary Figure 1**), estimated around 1 DNA copy/µL.


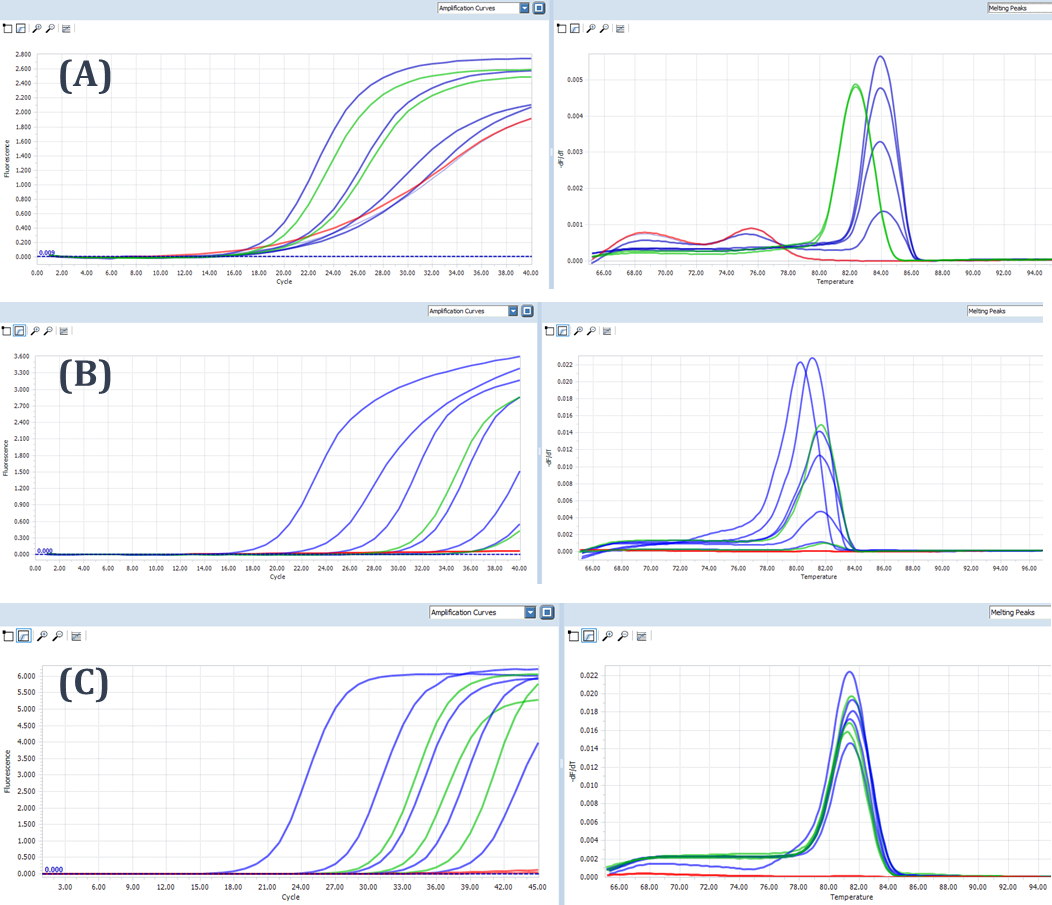
**Supplementary Figure 1** - Profiles of SYBR Green real-time (RT-)PCR results for sensitivity validation runs on **(A)** Avulavirus-Rubulavirus L gene target sequence (AVU-RUB-F1/AVU-RUB-R), **(B)** Coronavirus polymerase gene target sequence (AC-CoV-F/AC-CoV-R) and **(C)** *Chlamydia sp*. 23S rDNA target sequence (Ch23S-F/Ch23S-R).
For each figure, amplification curves on the left and melting peaks on the right. Red curves are from the negative controls. Other curves represent: **(A)** ten-fold dilutions (10 to 10^5^ copies/µL) of control plasmid with a NDV class I insert (in blue), and two dilutions of RNA from a cultivated NDV class II (in green); **(B)** ten-fold dilutions (10 to 10^6^ copies/µL) of control plasmid with a SARS-CoV-2 insert (in blue) and two dilutions of RNA from a cultivated vaccinal infectious bronchitis virus (in green); and **(C)** ten-fold dilutions (1 to 10^3^, and 10^5^ copies/µL) of control plasmid with a *Chlamydia psittaci* insert (in blue), and three duck swab samples that were consecutively sequenced as GR9 strain of *C. psittaci* (in green).
Thermocycling programs were ran on a LightCycler 96 thermocycler (Roche, Basel, Switzerland) following protocols described in **Supplementary Table 2**.

# Final protocols used in the study for molecular detection and identification of studied infectious agents

All reactions of real-time (RT-)PCR were processed on a LightCycler 96 thermocycler (Roche, Basel, Switzerland), and conventional (RT-)PCR were processed on a peqSTAR thermocycler (Avantor, Radnor, Pennsylvania, USA), following protocols described in **Supplementary Table 2**.

**Supplementary Table 2** – Description of real-time (RT-)PCR reagents and programs used for molecular screening and identification of infectious agents in the study.

| **Target agent** | **Aim of PCR** | **Target sequence (length)** | **Primer pair name** | **Primer pair sequence (5'-3')** | **Primer reference** |
| --- | --- | --- | --- | --- | --- |
| Avian Influenza Viruses (AIV) | Detection | M gene (240 bp) | M52C | CTT CTA ACC GAG GTC GAA ACG | [30,2add] |
|  |  |  | M253R | AGG GCA TTT TGG ACA AAK CGT CTA |  |
|  | **Reaction kit** | iTaq Universal SYBR green One-Step kit (Biorad, Hercules, CA, USA) | | | |
|  | **Reagent mix** | 2 µL RNA - 0.5 µL of each 10 µM primer – 5 µL of 2X iTaq reaction mix - 0.125 µL of iScript reverse transcriptase - 1.875 µL of PCR-grade water /10 µL total | | | |
|  | **Thermocycling program** | 10 min at 50°C – 1 min at 95°c - 40x(15 s at 95°C – 1 min at 60°C) - melting analysis following manufacturer’s instructions | | | |
|  | **Expected detection threshold (genetic copies/µL)** | 10 |  |  |  |
| AIV | Identification - run 1  (Sanger sequencing) | HA gene (∼1800 bp) | Bm-HA-1 | TAT TCG TCT CAG GGA GCA AAA GCA GGG G | [31] |
|  |  |  | Bm-NS-890R | ATA TCG TCT CGT ATT AGT AGA AAC AAG GGT GTT TT |  |
|  | **Reaction kit** | QIAGEN OneStep RT-PCR kit (Qiagen, Venlo, the Netherlands) | | | |
|  | **Reagent mix** | 2 µL RNA - 1.5 µL of each 10 µM primer – 5 µL of QIAGEN OneStep RT-PCR Buffer (5X) - 1µL of 10mM dNTP mix - 1µL of QIAGEN OneStep RT-PCR Enzyme Mix – 13 µL of PCR-grade water /25µL total | | | |
|  | **Thermocycling program** | 30 min at 50°C – 15 min at 95°C - 5x(30s at 95°C - 30s at 56°C - 1min at 72°C) - 30x(30 s at 95°C – 30 s at 54°C – 1 min at 72°C) - 5x(30 s at 95°C – 30 s at 52°C – 1 min at 72°C) - 5 min at 72°C | | | |
|  | **Expected detection threshold (genetic copies/µL)** | - |  |  |  |
| AIV | Identification - run 2  (Sanger sequencing) | HA gene (∼1800 bp) | Bm-HA-1 | TAT TCG TCT CAG GGA GCA AAA GCA GGG G | [31] |
|  |  |  | Bm-NS-890R | ATA TCG TCT CGT ATT AGT AGA AAC AAG GGT GTT TT |  |
|  | **Reaction kit** | KAPA Taq PCR kit (Kapa Biosystems, Wilmington, Massachusetts, USA) | | | |
|  | **Reagent mix** | 2 µL run 1 purified 1800 bp products – 2 µL of each 10 µM primer - 2.5 µL of KAPA Taq Buffer A (10X) - 0.5 µL of 10 mM dNTP mix - 0.1 µL of KAPA Taq DNA Polymerase (5 U/µL) - 15.9 µL of PCR-grade water /25 µL total | | | |
|  | **Thermocycling program** | 3 min at 95°C - 40x(30 s at 95°C – 30 s at 53°C - 1.5 min at 72°C) - 1 min at 72°C | | | |
|  | **Expected detection threshold (genetic copies/µL)** | - |  |  |  |

**Supplementary Table 2** – Continued

| **Target agent** | **Aim of PCR** | **Target sequence (length)** | **Primer pair name** | **Primer pair sequence (5'-3')** | **Primer reference** |
| --- | --- | --- | --- | --- | --- |
| AIV - H6 | Identification - run 3 | HA gene (240 bp) | H6-928 | CCA CAT GCC AGA CTA TTG CAG G | This study |
|  |  |  | H6-1251 | CGA CAG CTT CGA ATT GTG TGT TC |  |
|  | **Reaction kit** | iTaq Universal SYBR green One-Step kit (Biorad, Hercules, CA, USA) | | | |
|  | **Reagent mix** | 2µL RNA - 0.5 µL of each 10 µM primer – 5 µL of 2X iTaq reaction mix - 0.125 µL of iScript reverse transcriptase - 1.875 µL of PCR-grade water /10 µL total | | | |
|  | **Thermocycling program** | 10 min at 50°C – 1 min at 95°C - 40x(15 s at 95°C – 1 min at 60°C) - melting analysis following manufacturer’s instructions | | | |
|  | **Expected detection threshold (genetic copies/µL)** | 10 |  |  |  |
| Avulaviruses | Detection | L gene (200 bp) | AVU-RUB-F1 | GGT TAT CCT CAT TTI TTY GAR TGG ATH CA | [34] |
|  |  |  | AVU-RUB-R | GCA ATT GCT TGA TTI TCI CCY TGN AC |  |
|  | **Reaction kit** | iTaq Universal SYBR green One-Step kit (Biorad, Hercules, CA, USA) | | | |
|  | **Reagent mix** | 2 µL RNA - 0.6 µL of each 10 µM primer – 5 µL of 2X iTaq reaction mix - 0.125 µL of iScript reverse transcriptase - 1.675 µL of PCR-grade water /10 µL total | | | |
|  | **Thermocycling program** | 10 min at 50°C – 1 min at 95°C - 40x(15 s at 95°C – 15 s at 55°C – 1 min at 60°C) - melting analysis following manufacturer’s instructions | | | |
|  | **Expected detection threshold (genetic copies/µL)** | 10² |  |  |  |
| Avulaviruses | Identification  (Sanger sequencing) | L gene (200 bp) | AVU-RUB-F2 | ACA CTC TAT GTI GGI GAI CCN TTY AAY CC | [34] |
|  |  |  | AVU-RUB-R | GCA ATT GCT TGA TTI TCI CCY TGN AC |  |
|  | **Reaction kit** | KAPA Taq PCR kit (Kapa Biosystems, Wilmington, Massachusetts, USA) | | | |
|  | **Reagent mix** | 2 µL purified detection run products - 1.5 µL of each 10 µM primer - 2.5 µL of KAPA Taq Buffer A (10X) - 0.5 µL of 10 mM dNTP mix - 0.1 µL of KAPA Taq DNA Polymerase (5 U/µL) - 11.9 µL of PCR-grade water /20 µL total | | | |
|  | **Thermocycling program** | 3 min at 95°C - 40x(30 s at 95°C – 30 s at 53°C - 1.5 min at 72°C) - 1 min at 72°C | | | |
|  | **Expected detection threshold (genetic copies/µL)** | - |  |  |  |

**Supplementary Table 2** – Continued

| **Target agent** | **Aim of PCR** | **Target sequence (length)** | **Primer pair name** | **Primer pair sequence (5'-3')** | **Primer reference** |
| --- | --- | --- | --- | --- | --- |
| Coronaviruses | Detection | polymerase gene (600 bp) | AC-CoV-F | GGT TGG GAT TAT CCW AAR TGT G | [35] |
|  |  |  | AC-CoV-R | TGY TGT GAR CAA AAY TCR TG |  |
|  | **Reaction kit** | iTaq Universal SYBR green One-Step kit (Biorad, Hercules, CA, USA) | | | |
|  | **Reagent mix** | 2 µL RNA - 0. 6µL of each 10 µM primer – 5 µL of 2X iTaq reaction mix - 0.125 µL of iScript reverse transcriptase - 1.675 µL of PCR-grade water /10 µL total | | | |
|  | **Thermocycling program** | 10 min at 50°C – 1 min at 95°C - 40x(15 s at 95°C – 15 s at 50°C – 1 min at 60°C) - melting analysis following manufacturer’s instructions | | | |
|  | **Expected detection threshold (genetic copies/µL)** | 10 |  |  |  |
| Coronaviruses | Identification  (Sanger sequencing) | polymerase gene (600 bp) | AC-CoV-F | GGT TGG GAT TAT CCW AAR TGT G | [35] |
|  |  |  | AC-CoV-R | TGY TGT GAR CAA AAY TCR TG |  |
|  | **Reaction kit** | KAPA Taq PCR kit (Kapa Biosystems, Wilmington, Massachusetts, USA) | | | |
|  | **Reagent mix** | 2 µL purified detection run products - 1.5 µL of each 10 µM primer - 2.5 µL of KAPA Taq Buffer A (10X) - 0.5 µL of 10 mM dNTP mix - 0.1 µL of KAPA Taq DNA Polymerase (5 U/µL) - 16.9 µL of PCR-grade water /25 µL total | | | |
|  | **Thermocycling program** | 3 min at 95°C - 40x(30 s at 95°C – 30 s at 53°C - 1.5 min at 72°C) - 1 min at 72°C | | | |
|  | **Expected detection threshold (genetic copies/µL)** | - |  |  |  |
| *Chlamydia* sp. | Detection | 23S rDNA (180 bp) | Ch23S-F | CTG AAA CCA GTA GCT TAT AAG CGG T | [36] |
|  |  |  | Ch23S-R | ACC TCG CCG TTT AAC TTA ACT CC |  |
|  | **Reaction kit** | LightCycler SYBR Green I Master reaction mix (Roche, Basel, Switzerland) | | | |
|  | **Reagent mix** | 2 µL DNA - 0.4 µL of each 10 µM primer – 10 µL of SYBR Green I Master - 7.2 µL of PCR-grade water /20 µL total | | | |
|  | **Thermocycling program** | 5 min at 95°C - 45x(10 s at 95°C – 15 s at 55°C – 15 s at 72°C) - melting analysis following manufacturer’s instructions | | | |
|  | **Expected detection threshold (genetic copies/µL)** | 1 |  |  |  |

# 2add. Fouchier RAM, Bestebroer TM, Herfst S, Van Der Kemp L, Rimmelzwaan GF, Osterhaus ADME (2000) Detection of influenza A viruses from different species by PCR amplification of conserved sequences in the matrix gene. J Clin Microbiol 38:4096–4101
