# Supplementary material for: Limited transmission of avian influenza viruses, avulaviruses, coronaviruses and Chlamydia sp. at the interface between wild birds and a free-range duck farm
Source: Vet Res. 2025 Feb 8;56:36. doi: 10.1186/s13567-025-01466-3 (PMC11806813; doi:10.1186/s13567-025-01466-3)
Supplement: Supplementary file 4 — Additional file 4. Description of positive samples for all four infectious agents. [file 13567_2025_1466_MOESM4_ESM.docx]

***Description of positive samples for all four infectious agents***

**Supplementary Table 3.** Description of positive samples for all four infectious agents.

| **Infectious agent detected** | **Month (week) and year of sampling** | **Sample type** | **Wild bird group (species if known) or duck flock identification (based on those of Table 1)**  (small letters and colors refer to same wild bird or flock) | **Positive detection test and its result**  (Ct for PCR, competition percentage for ELISA) | **Infectious agent identification** (identified by sequence, PCR, HRM-analysis, or result provided by the reference laboratory)  (identified genotypes are mentioned) |
| --- | --- | --- | --- | --- | --- |
| **AIV** | June (24) 2019 | Duck flock (regulatory) | C; D | PCR | H7N3 (reference laboratory) |
|  | July (30) 2019 | Wild (oropharyngeal) | SYL (*Sylvia atricapilla*) | PCR (33.54) | - |
|  | August (35) 2019 | Wild (cloacal) | MUS | PCR (33.65) | - |
|  | August (35) 2019 | Wild (oropharyngeal) | PASD (*Passer domesticus*) | PCR (36.98) | - |
|  | August (35) 2019 | Wild (blood) | SYL (*Sylvia atricapilla*) | ELISA (46.67%) | - |
|  | August (35) 2019 | Wild (blood) | MUS (*Luscinia megarhynchos*) | ELISA (22.98%) | - |
|  | August (35) 2019 | Wild (blood) | MUS (*Ficedula hypoleuca*) | ELISA (43.92%) | - |
|  | October (43) 2019 | Wild (cloacal) | MOT (*Motacilla alba*) **i** | PCR (33.04) | - |
|  | October (43) 2019 | Wild (blood) | MOT (*Motacilla alba*) | ELISA (33.43%) | - |
|  | October (43) 2019 | Wild (blood) | SYL (*Sylvia atricapilla*) | ELISA (49.24%) | - |
|  | October (43) 2019 | Wild (blood) | SYL (*Sylvia atricapilla*) | ELISA (29.09%) | - |
|  | October (43) 2019 | Wild (blood) | TUR (*Turdus philomelos*) | ELISA (48.14%) | - |
|  | November (48) 2019 | Wild (cloacal) | ANT (*Anthus sp*.) | PCR (34.00) | - |
|  | November (48) 2019 | Wild (oropharyngeal) | PASD (*Passer domesticus*) | PCR (33.36) | - |
|  | December (52) 2019 | Duck flock (cloacal) | K | PCR (34.31) | H6 (sequence) |
|  | January (05) 2020 | Environment | M | PCR (32.86) | - |
|  | January (05) 2020 | Environment | N | PCR (33.95) | H6 (PCR) |
|  | January (05) 2020 | Wild (oropharyngeal) | SYL (*Sylvia atricapilla*) | PCR (35.48) | - |
|  | January (05) 2020 | Wild (oropharyngeal) | FRI (*Fringilla sp*.) | PCR (36.17) | - |
|  | January (05) 2020 | Wild (blood) | PASM (*Passer montanus*) **e** | ELISA (39.89%) | - |
|  | February (08) 2020 | Duck flock (cloacal) | M | PCR (32.42) | H6 + H11 (sequence) |
|  | February (08) 2020 | Duck flock (regulatory) | N | PCR | not H5 or H7 (reference laboratory) |
|  | February (09) 2020 | Environment | M | PCR (32.98) | H6 (PCR) |
|  | February (09) 2020 | Wild (blood) | PASM (*Passer montanus*) **e** | ELISA (42.10%) | - |
|  | May (22) 2020 | Wild (blood) | MUS (*Luscinia megarhynchos*) | ELISA (10.18%) | - |
|  | May (22) 2020 | Wild (blood) | SYL (*Sylvia atricapilla*) | ELISA (38.53%) | - |
|  | May (22) 2020 | Wild (blood) | SYL (*Sylvia atricapilla*) | ELISA (43.04%) | - |
|  | May (22) 2020 | Wild (blood) | TUR (*Turdus philomelos*) | ELISA (30.10%) | - |
|  | November (46) 2020 | Environment | T | PCR (31.81) | H6 (sequence) |
|  | November (46) 2020 | Environment | U | PCR (34.04) | H6 (sequence) |
|  | November (46) 2020 | Wild (blood) | MUS (*Erithacus rubecula*) | ELISA (38.45%) | - |
|  | November (46) 2020 | Wild (blood) | MUS (*Erithacus rubecula*) | ELISA (36.44%) | - |
|  | November (46) 2020 | Wild (blood) | PASD (*Passer domesticus*) | ELISA (46.96%) | - |
|  | November (46) 2020 | Wild (blood) | PRU (*Prunella modularis*) | ELISA (32.37%) | - |
|  | November (46) 2020 | Wild (blood) | PRU (*Prunella modularis*) | ELISA (26.78%) | - |
|  | November (46) 2020 | Wild (blood) | TUR (*Turdus philomelos*) | ELISA (45.85%) | - |
|  | December (51) 2020 | Environment | T | PCR (34.84) | H6 (PCR) |
|  | December (51) 2020 | Environment | V | PCR (35.16) | H6 (PCR) |
|  | December (51) 2020 | Environment | W | PCR (31.03) | H6 (PCR) |
|  | December (51) 2020 | Wild (blood) | MOT (*Motacilla alba*) | ELISA (10.22%) | - |
|  | December (51) 2020 | Wild (blood) | MOT (*Motacilla alba*) | ELISA (10.05%) | - |
|  | December (51) 2020 | Wild (blood) | MOT (*Motacilla alba*) | ELISA (9.37%) | - |
|  | February (09) 2021 | Wild (feces) | BUB (*Bubulcus ibis*) | PCR (35.50) | H5 low pathogenic (sequence) |
|  | February (09) 2021 | Wild (blood) | ACC (*Accipiter nisus*) | ELISA (10.54%) | - |
|  | February (09) 2021 | Wild (blood) | MOT (*Motacilla alba*) | ELISA (15.81%) | - |
|  | February (09) 2021 | Wild (blood) | PRU (*Prunella modularis*) | ELISA (38.84%) | - |
| **Avulaviruses** | January (05) 2020 | Wild (blood) | TUR (*Turdus merula*) **f** | ELISA (69.59%) | - |
|  | February (09) 2020 | Wild (blood) | TUR (*Turdus merula*) **f** | ELISA (56.02%) | - |
|  | December (51) 2020 | Wild (blood) | ANT (*Anthus pratensis*) | ELISA (41.38%) | - |
|  | December (51) 2020 | Wild (blood) | MOT (*Motacilla alba*) | ELISA (65.53%) | - |
| **Coronaviruses** | October (43) 2019 | Environment | H | PCR (33.30) | Duck igacovirus (sequence) |
|  | November (45) 2019 | Duck flock (cloacal) | G | PCR (31.83) | Duck igacovirus (sequence) |
|  | December (52) 2019 | Duck flock (cloacal) | K | PCR (38.28) | Duck igacovirus (sequence) |
|  | November (46) 2020 | Environment | T | PCR (-) | - |
|  | November (46) 2020 | Environment | V | PCR (38.52) | - |
| **Chlamydia** | July (30) 2019 | Wild (oropharyngeal) | PASM (*Passer montanus*) | PCR (37.00) | - |
|  | August (35) 2019 | Wild (oropharyngeal & cloacal) | AEG (*Phylloscopus collybita*) | PCR (35.34) | avian *C. abortus* (ST329) (sequence) |
|  | August (35) 2019 | Wild (cloacal) | SYL (*Sylvia communis*) | PCR (27.00) | avian *C. abortus* (ST330) (sequence) |
|  | October (43) 2019 | Wild (cloacal) | MOT (*Motacilla alba*) | PCR (40.00) | - |
|  | October (43) 2019 | Wild (oropharyngeal) | MOT (*Motacilla alba*) | PCR (39.90) | - |
|  | October (43) 2019 | Wild (oropharyngeal) | SYL (*Sylvia atricapilla*) | PCR (37.25) | - |
|  | December (52) 2019 | Duck flock (oropharyngeal & cloacal) | K | PCR (32.66) | *C. psittaci* (Group II_Duck) (HRM) |
|  | January (05) 2020 | Environment | M | PCR (34.79) | *C. psittaci* (PCR) |
|  | January (05) 2020 | Environment | N | PCR (33.81) | *C. psittaci* (Group II_Duck) (HRM) |
|  | February (08) 2020 | Duck flock (cloacal) | M | PCR (35.32) | *C. psittaci* (Group II_Duck) (HRM) |
|  | February (09) 2020 | Environment | O | PCR (29.72) | *C. psittaci* (Group II_Duck) (HRM) |
|  | February (09) 2020 | Wild (oropharyngeal) | AEG | PCR (37.90) | - |
|  | February (09) 2020 | Wild (cloacal) | FRI (*Fringilla coelebs*) | PCR (35.50) | avian *C. psittaci* (PCR) |
|  | November (46) 2020 | Environment | T | PCR (32.92) | avian *C. abortus* (PCR) |
|  | November (46) 2020 | Environment | U | PCR (35.74) | avian *C. abortus* (PCR) |
|  | November (46) 2020 | Wild (oropharyngeal) | AEG (*Phylloscopus collybita*) | PCR (34.81) | avian *C. abortus* (ST329) (sequence) |
|  | December (51) 2020 | Environment | T | PCR (36.59) | avian *C. abortus* (PCR) |
|  | December (51) 2020 | Environment | U | PCR (34.59) | *C. psittaci* (Group II_Duck) (HRM) |
|  | December (51) 2020 | Environment | V | PCR (33.47) | *C. psittaci* (Group II_Duck) (HRM) |
|  | December (51) 2020 | Environment | W | PCR (34.41) | avian *C. abortus* (PCR) |
