# Supplementary material for: Limited transmission of avian influenza viruses, avulaviruses, coronaviruses and Chlamydia sp. at the interface between wild birds and a free-range duck farm
Source: Vet Res. 2025 Feb 8;56:36. doi: 10.1186/s13567-025-01466-3 (PMC11806813; doi:10.1186/s13567-025-01466-3)
Supplement: Supplementary file 5 — Additional file 5. Phylogeny of the identified Chlamydia sp. [file 13567_2025_1466_MOESM5_ESM.docx]

Phylogeny of identified Chlamydia sp.


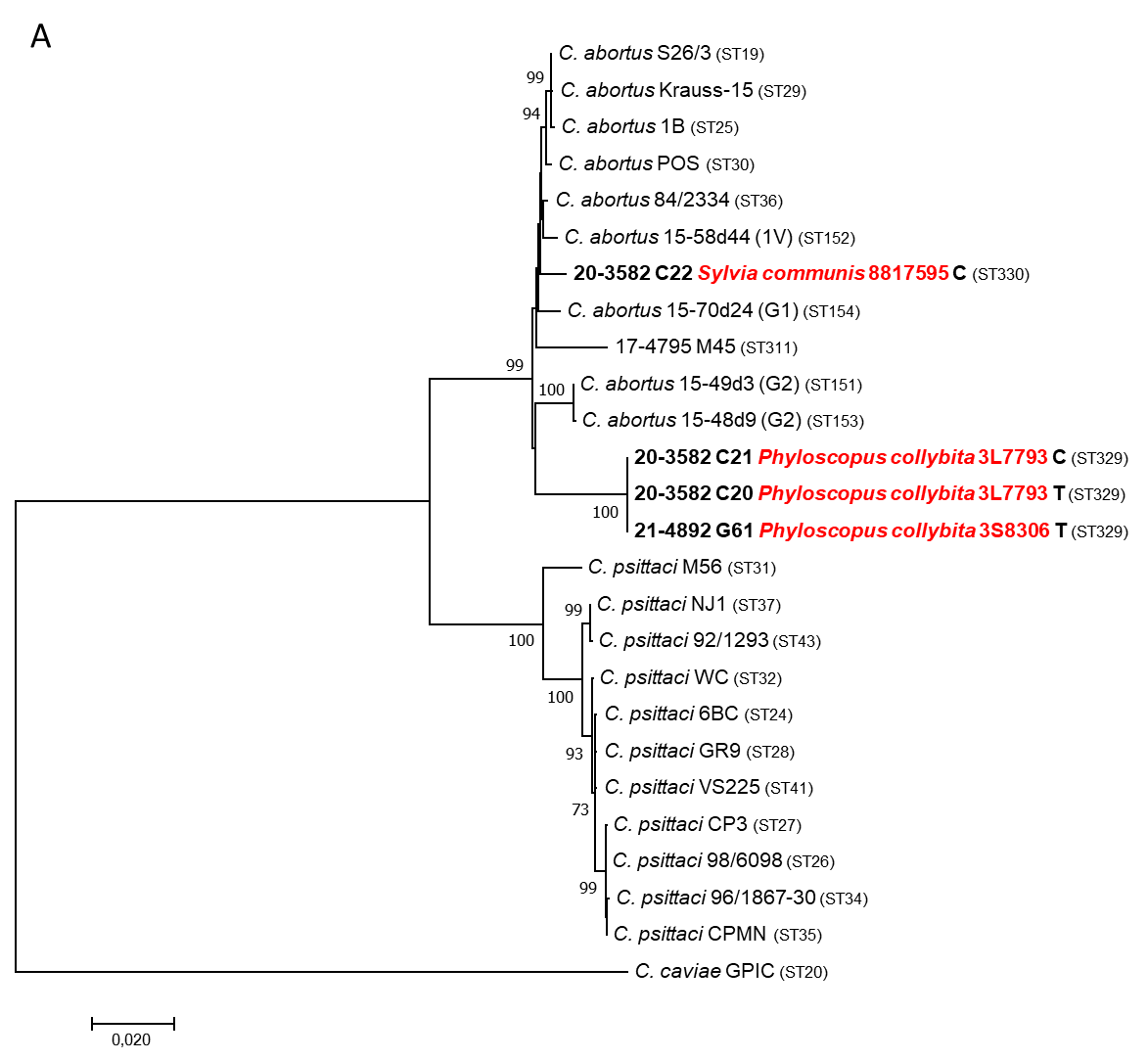


**A**


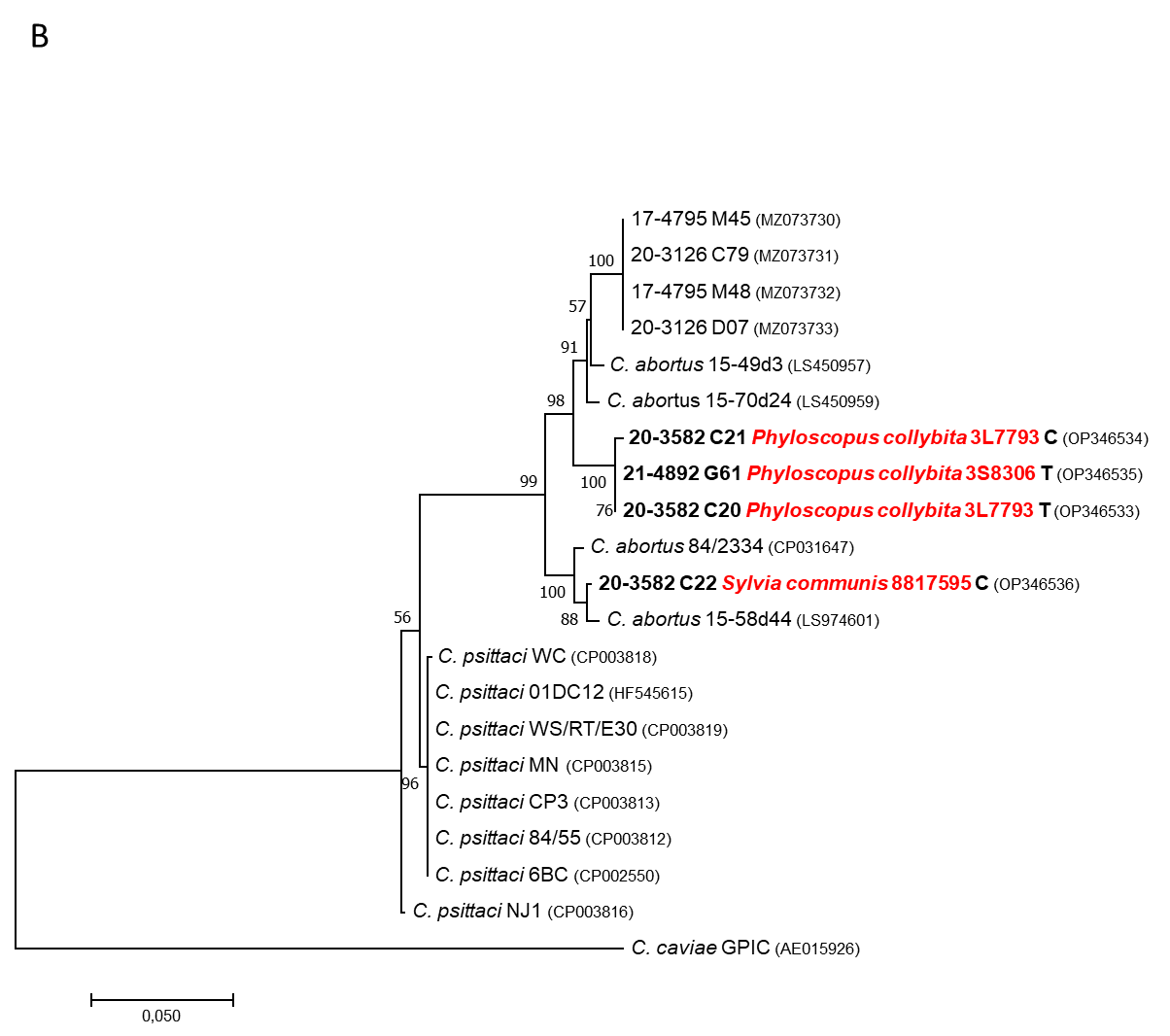


**B**

**Supplementary Figure 2.** Phylogenetic analyses of (**A**) concatenated sequences of seven MLST housekeeping gene fragments (*eno*A, *fum*C, *gat*A, *gid*A, *hem*N, *hfl*X, and *opp*A) and of (**B**) the plasmid sequences (707 bp) for four wild bird specimens (20-3582 C20, 20-3582 C21, 20-3582 C22, 21-4892 G61) and representative sequences.
Briefly, DNA sequences were aligned by using ClustalW. Then the best Maximum Likelihood (ML) model with the lowest Bayesian Information Criterion (BIC) were applied according to MEGA7 software [1]. Trees were then inferred by using the ML method based on the Tamura 3-parameter model. Trees with the highest log-likelihood are shown. The percentage of replicate trees in which the associated taxa clustered together is shown next to the branches (1000 bootstraps, only values above 50% are shown). The trees are drawn to scale, with branch lengths measured in the number of substitutions per site. Evolutionary analyses were conducted in MEGA7 [3add].

3add. Kumar S, Stecher G, Tamura K (2016) MEGA7: molecular evolutionary genetics analysis version 7.0 for bigger datasets. Mol Biol Evol 33:1870–1874
