# Supplementary material for: Limited transmission of avian influenza viruses, avulaviruses, coronaviruses and Chlamydia sp. at the interface between wild birds and a free-range duck farm
Source: Vet Res. 2025 Feb 8;56:36. doi: 10.1186/s13567-025-01466-3 (PMC11806813; doi:10.1186/s13567-025-01466-3)
Supplement: Supplementary file 6 — Additional file 6. Population sensitivity for each time of wild bird sampling. [file 13567_2025_1466_MOESM6_ESM.docx]

***Population sensitivity for each time of wild bird sampling***

**Supplementary Table 4.** Population sensitivity for each time of wild bird sampling.
For each sample size, sensitivity calculation is based for each test on its estimated individual sensitivity (PCR 0.99, ELISA 0.9) and specificity (PCR 1, ELISA 0.9), with a confidence level of 95%.

| **Year** | **Month** | **Week** | **Swabs** | **Feces**  **(cattle egrets)** | **Sample size swabs/feces** | **Population sensitivity PCR** | **Sample size**  **blood** | **Population sensitivity ELISA** |
| --- | --- | --- | --- | --- | --- | --- | --- | --- |
| **2019** | **July** | **30** | 184 |  | 184 | **1.6%** | 0 | **-** |
|  | **August** | **35** | 256 |  | 256 | **1.2%** | 121 | **2.7%** |
|  | **October** | **43** | 240 | 30 | 270 | **1.1%** | 142 | **2.3%** |
|  | **November** | **48** | 184 | 30 | 214 | **1.4%** | 75 | **4.4%** |
| **2020** | **January** | **5** | 225 |  | 225 | **1.3%** | 112 | **2.9%** |
|  | **February** | **9** | 141 |  | 141 | **2.1%** | 88 | **3.7%** |
|  | **May** | **22** | 168 |  | 168 | **1.8%** | 140 | **2.4%** |
|  | **November** | **46** | 225 |  | 225 | **1.3%** | 147 | **2.2%** |
|  | **December** | **51** | 47 | 70 | 117 | **2.6%** | 40 | **8.0%** |
| **2021** | **February** | **6** |  | 25 | 25 | **11.4%** | 0 | **-** |
|  |  | **9** | 51 | 50 | 101 | **3.0%** | 40 | **8.0%** |
|  | **March** | **12** | 10 |  | 10 | **26.0%** | 5 | **50.0%** |
